# DJ-X-013 reduces LPS-induced inflammation, modulates Th17/myeloid-derived suppressor cells, and alters NF-κB expression to ameliorate experimental colitis

**DOI:** 10.1016/j.biopha.2024.117379

**Published:** 2024-09-09

**Authors:** Mousumi Mandal, Ahmed Rakib, Md Abdullah Al Mamun, Santosh Kumar, Frank Park, Dong-Jin Hwang, Wei Li, Duane D. Miller, Udai P. Singh

**Affiliations:** Department of Pharmaceutical Sciences, College of Pharmacy, The University of Tennessee Health Science Center, Memphis, TN 38163, USA

**Keywords:** Inflammation, Colitis, Th17, Cytoskeletal protein, MDSCs

## Abstract

**Scope:**

Inflammatory bowel disease (IBD) is a chronic intestinal inflammatory condition of unknown etiology, although recent evidence suggests that it is caused by an excessive immune response to mucosal antigens. We determined the anti-inflammatory properties of novel compound DJ-X-013 *in vitro* in lipopolysaccharide (LPS)-induced macrophages and in an *in vivo* dextran sodium sulfate (DSS)-induced model of colitis.

**Methods and results:**

To evaluate the anti-inflammatory properties of DJ-X-013, we used LPS-activated RAW 264.7 macrophages *in vitro* and a DSS-induced experimental model of colitis *in vivo*. We examine cellular morphology, and tissue architecture by histology, flow cytometry, RT-qPCR, multiplex, and immunoblot analysis to perform cellular and molecular studies. DJ-X-013 treatment altered cell morphology and expression of inflammatory cytokines in LPS-activated macrophages as compared to cells treated with LPS alone. DJ-X-013 also impeded the migration of RAW 264.7 macrophages by modulating cytoskeletal organization and suppressed the expression of NF-κB and inflammatory markers as compared to LPS alone. DJ-X-013 treatment improved body weight, and colon length and attenuated inflammation in the colon of DSS-induced colitis. Intriguingly, DSS-challenged mice treated with DJ-X-013 induced the numbers of myeloid-derived suppressor cells (MDSCs), dendritic cells (DCs), and natural killer T cells (NKT) in the colon lamina propria (LP) relative to DSS. DJ-X-013 also reduced the influx of neutrophils, TNF-α producing macrophages, restricted the number of Th17 cells, and suppressed inflammatory cytokines and NF-κB in the LP relative to DSS.

**Conclusion:**

DJ-X-013 is proposed to be a therapeutic strategy for ameliorating inflammation and experimental colitis.

## 1. Introduction

Inflammation is an evolutionarily conserved process that serves as the body’s defense program against pathogens. This process has a defined beginning and end, culminating in a prompt termination sequence. However, if the inflammation process extends for a prolonged period or with a higher-than-normal magnitude, it can cause tissue and organ damage, delay wound healing, and pave the way for the development of diseases such as cardiovascular disease and cancer [1]. Notably, chronic inflammation increases the likelihood of developing autoimmune diseases including rheumatoid arthritis (RA), multiple sclerosis (MS), systemic lupus erythematosus (SLE), type 1 diabetes mellitus (T1DM), psoriasis, psoriatic arthritis, and inflammatory bowel disease (IBD) [2]. The incidence of autoimmune diseases can be as high as one individual in ten, meaning that these diseases represent a constant challenge for society and a significant burden on healthcare systems [3]. IBDs, including Crohn’s disease (CD) and ulcerative colitis (UC), are highly prevalent (>0.3 %) in developed countries [4], and affect approximately 6.8 million individuals globally [5]. IBDs are characterized by aberrant inflammatory responses in the intestine and destruction of the epithelial barrier as a result of environmental factors, dysbiosis, genetic predisposition, diet, and lifestyle [6]. While the precise etiology of IBD remains unclear, experimental studies suggest a role for dysregulation in the mucosal immune system in the pathogenesis of IBDs [7–9]. IBD is normally associated with chronic inflammation induced by infiltration of immune cells in the colon, including macrophages, neutrophils, T helper 17 (Th17) cells, and type 1 T helper (Th1) cells, resulting in the secretion of the proinflammatory cytokines interleukin 6 (IL-6), tumor necrosis factor-alpha (TNF-α), and interferon-gamma (IFN-γ) [10–12]. While existing therapeutic approaches lessen the severity of IBD, they also generate lots of side effects and can weaken the immune system to the extent that the disease can become refractory to treatment in some patients [13]. To circumvent these difficulties, an estimated 50 % of IBD patients resort to alternative medications, including prebiotics, probiotics, and medical cannabis, for the relief of their symptoms [14]. Thus, it is essential to continue the search for new safe, cost-effective therapeutic approaches that will restore immune homeostasis and prevent the symptoms of IBD. To this end, we used computer-aided prediction tools to design the compound DJ-X-013, synthesized it, and tested its anti-inflammatory properties using *in vitro* and *in vivo* analyses.

Cytoskeletal proteins, primarily actin and tubulin, serve to maintain cellular morphology and function [15]. Highly dynamic tubulin microtubules (MTs) located in the cell cytoplasm regulate cell division, motility, morphology, organization, and internal cellular transport [16]. MT dysfunction induces a hyper-stimulatory cellular response and has been implicated as a mediator of inflammation in multiple diseases [17]. In response to suppression of MT dynamics in human breast cancer cell line MCF-7, MTs associated with transcription factor nuclear factor kappa-light-chain-enhancer of activated B cells (NF-κB) stimulate apoptosis [18]. The cytoskeleton also acts as a central regulator of innate immune cells [19], and plays a role in immune synapse and polarization during T-cell activation [20]. In ciliated cells such as those in the colon, post-translational modification of tubulin by glycylation regulates the length of primary cilia. In mammals, MT glycylation is catalyzed by tubulin tyrosine ligase-like (TTLL) glycyases, which control the proliferation of ciliated colon epithelial cells and play an essential role in the development of colon cancer [21]. Since IBD tissue injury, mucosal disruption, and symptomatic flare-ups are associated with oxidant-induced disruption of the cytoskeleton [22], we wished to determine the effect of DJ-X-013 treatment on the cytoskeleton in RAW 264.7 macrophages in a lipopolysaccharide (LPS)-induced model of inflammation. Specifically, we set out to determine whether DJ-X-013 modulates cell morphology and migration through cytoskeletal reorganization to suppress NF-κB pathways during an LPS-induced inflammatory response.

In experimental animal models of IBD, its progression results in part from dysregulation of macrophages, natural killer T (NKT) cells, and T lymphocytes, including Th17 helper T cells that secrete cytokines IL-17 and IL-22 [23,24]. In CD patients, the number of infiltrating Th17 cells increased relative to healthy patients [10,25], and dextran sodium sulfate (DSS)-induced colitis in mice is characterized by a further increase in the number of Th17 cells [26,27]. Since IL-17 is important for both inducing and abating chemically induced colitis in mice [28,29], we felt it critical to determine whether DJ-X-013 suppressed the Th17 response in mice with DSS-induced experimental colitis.

Macrophages, which are prominent in active IBD, play an important role in the pathogenesis of Th1-mediated IBD [30–32]. During the early stages of differentiation, the cell population known as heterogeneous myeloid-derived suppressor cells (MDSCs) serve as the precursors of macrophages, granulocytes, and dendritic cells (DCs) when stimulated with selected sets of cytokines [33]. MDSCs play a key role in the recruitment of other immune cells [34], serve as potent suppressors of the immune response [35], and expand during chronic inflammatory pathologies to serve as crucial players in the prevention of diseases [36]. In mice with intestinal inflammation, the frequency of MDSCs increases significantly and these cells suppress T-cell activation [37,38]. The frequency of MDSCs also increases in the peripheral blood of patients with active colitis, perhaps serving to halt the development of more severe and possibly fatal colitis. Together, these studies highlighted the role of MDSCs as the basis of a previously unexplored immune regulatory mechanism in IBD that alters the frequency of macrophages and other inflammatory cells in DSS-induced colitis in mice. Thus, we determined whether DJ-X-013 modulated the frequency and ability of MDSCs to mediate the function of neutrophils, DCs, and T cells in the colon. We also examined its effect on the intricate network of cytokines, chemokines, and transcription factors involved in chronic inflammation and colitis, including TNF-α, IL-6, IFN-γ, IL-1β, C-X-C motif chemokine receptor 3 (CXCR3), signal transducer and activator of transcription (STAT3), NF-κB. Thus, the present study explored whether DJ-X-013 can be developed as a novel therapeutic agent for the treatment of colitis and other inflammatory diseases.

## 2. Materials and methods

### 2.1. Computer-aided evaluation of DJ-X-013 properties

**Fig. 1.**
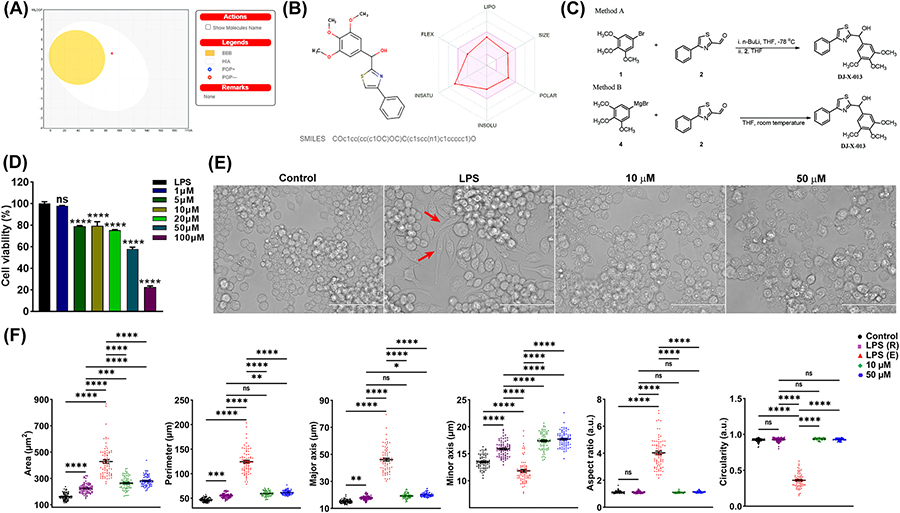
Formulation and synthesis of DJ-X-013 and effects of DJ-X-013 on cell viability and morphology of RAW 264.7 cells under *in vitro* LPS stimulation. (A) BOILED-Egg model for prediction of passive gastrointestinal (GI) adsorption and brain penetration for DJ-X-013, where the round, yellow “yolk” represents the BBB permeation region, the white oval represents the human intestinal absorption (HIA) region, and the gray rectangle represents the low absorption and limited brain permeation region. (B) Graphical output of the ADME prediction calculated for DJ-X-013 by SwissADME [39]. The pink shaded area represents the physicochemical space that is suitable for oral bioavailability, while DJ-X-013 is shown as a red line, calculated from its physical properties, which were assessed based on its predicted lipophilicity (LIPO), size (SIZE), polarity (POLAR), insolubility (INSOLU), insaturation (INSATU), and flexibility (FLEX). (C) Two alternative methods were used to synthesize DJ-X-013, method A (top panel) from 5-bromo-1,2,3-trimethoxybenzene (compound 1) and 4-phenylthiazole-2-carbaldehyde (compound 2) and method B (bottom panel) from 3,4,5-trimethoxyphenylmagnesiumbromide (compound 4) and compound 2. (D) Evaluation of cell viability by MTT assay after *in vitro* LPS stimulation in the absence or presence of DJ-X-013 treatment. Statistical analysis was performed using one-way ANOVA followed by Dunnett’s post hoc test; n = 3 per group. (E) Cell morphology after treatment with LPS or LPS plus 10 μM or 50 μM DJ-X-013 was determined by phase contrast microscopy (scale bar 100 μm). Elongated cells visible after treatment with LPS alone are indicated by red arrows. (F) Morphometric analysis of untreated cells and cells treated with LPS alone or LPS + DJ-X-013 (10 or 50 μM) was performed on phase contrast images using ImageJ software (NIH). The parameters examined were area (μm^2^), perimeter (μm), major axis (μm), minor axis (μm), aspect ratio (arbitrary units), and circularity (arbitrary units). Statistical analysis was performed using one-way ANOVA followed by Tukey’s multiple comparison post hoc tests; n = 75 for each group and/or cell type. LPS treatment alone resulted in a round - LPS (R) or elongated - LPS (E) cell morphology, while control cells and those in the presence of 10 or 50 μM DJ-X-013 were mostly round, suggesting that DJ-X-013 treatment either induces elongated cells to round cells reversion or abolishes the elongated cells from the population. Data are presented as mean values ± SEM, ns p > 0.05; *p < 0.05; **p < 0.01; ***p < 0.001; ****p < 0.0001.

We investigated the new nonsteroidal, thiazole-based compound DJ-X-013, which we expected to have anti-inflammatory properties like other thiazole-based compounds. DJ-X-013 could lead to the development of a safe new anti-inflammatory agent based on our computer-aided predictability analysis. We first evaluated the molecule DJ-X-013 using the computational methods provided by the SwissADME program (absorption, distribution, metabolism, and excretion) [39], calculating the expected physical properties (ADME) and other drug-likenesses of DJ-X-013 for use in pharmaceutical design (Fig. 1A and 1B).

### 2.2. Synthesis of (4-phenylthiazol-2-yl) (3,4,5-trimethoxyphenyl) methanol (DJ-X-013)

We synthesized DJ-X-013 using two alternative methods, A and B, as shown in Fig. 1C. In method A, a solution of n-butyllithium in hexane (2.5 M, 1.2 mL, 3 mmol) was added dropwise to a solution of 5-bromo-1,2,3-trimethoxybenzene (**compound 1**; 741 mg, 3 mmol) in tetrahydrofuran (THF; 15 mL) under nitrogen at −78°C. After 10 min., a pre-cooled (−78°C) solution of 4-phenylthiazole-2-carbaldehyde (**compound 2**; 568 mg, 3 mmol) in THF (10 mL) was added via a double-tipped needle. After stirring the mixture at −78° C for 30 min, the cooling bath was removed and the mixture was allowed to reach ambient temperature over 30 min. The solvent was removed *in vacuo*, the residue was partitioned between ethyl acetate and water, and the phases were separated. The organic layer was washed with brine, dried (MgSO_4_), and the solvent was removed *in vacuo*. The crude product was purified by column chromatography on silica gel using hexane/ethyl acetate (70:30, v/v) as the eluent to yield the designed compound as a yellow solid (DJ-X-013). Yield = 74 %. Purity (LC, *t*_min_ = 3.59) 99.70 %; UV λmax 254.45 nm, MS (ESI) m/z 358.10 [M + H] ^+^; LCMS (ESI) *m/z* calculated for C_19_H_19_NO_4_S 358.1113 [M + H] ^+^; found: 358.1104 [M + H] ^+^, 340.1000 [M − H_2_O + H] ^+^; ^1^H NMR (CDCl_3_, 400 MHz) δ 7.89 (d, *J* = 7.2 Hz, 2 H, ArH), 7.45 (d, *J* = 7.2 Hz, 2 H, ArH), 7.42 (s, 1 H, ArH), 7.34 (t, *J* = 7.6 Hz,1 H, ArH), 6.76 (s, 2 H, ArH), 6.03 (s, 1 H, C*H*-OH), 3.86 (s, 6 H, (OCH_3_)_2_), 3.85 (s, 3 H, OCH_3_), 1.68 (bs, 1 H, O*H*); ^13^C NMR (CDCl_3_, 100 MHz) δ 173.55, 154.92, 153.46 (2 C), 137.91, 136.79, 134.10, 128.80 (2 C), 128.30, 126.32 (2 C), 103.51 (2 C), 73.92, 60.87, 56.15 (2 C).

Alternatively, in method B, a solution of 4-phenylthiazole-2-carbaldehyde (**compound 2**; 0.568 g, 3 mmol) in 20 mL THF was slowly added to a solution of 3,4,5-trimethoxyphenylmagnesiumbromide (**compound 4**; 0.5 N, 6.0 mL, 3 mmol) in THF at 0 °C. The mixture was stirred for 2 h until the starting aldehyde (**compound 2**) disappeared, then quenched with saturated NH_4_Cl, extracted with ethyl ether, and dried with anhydrous MgSO_4_. The solvent was removed under reduced pressure to yield a crude product that was purified by column chromatography to obtain a 72.9 % yield of pure target compound DJ-X-013.

### 2.3. Cell culture with LPS and DJ-X-013 treatment

Mouse macrophage RAW264.7 cells (Cat. no. TIB-71, ATCC, USA) were cultured in Dulbecco’s modified essential medium (DMEM) (Cat. no. 10–027-CV, Corning, NY) supplemented with 10 % fetal bovine serum (FBS) and 1 % penicillin/streptomycin antibiotic solution, and maintained at 37°C, 5 % CO_2_ in an incubator as described previously [40]. Cells were seeded in appropriate dishes and 24 h later were treated with 100 ng/mL LPS (Cat. no. L4391, Millipore Sigma, St. Louis, MO) and 10 μM or 50 μM DJ-X-013 for 24 h. The groups analyzed were as follows: cells in the control group were incubated without LPS or DJ-X-013, cells in the LPS group were treated with only LPS, cells in the 10 μM group were treated with LPS + 10 μM DJ-X-013, and cells in the 50 μM group were treated with LPS + 50 μM of DJ-X-013.

### 2.4. Cell viability assay

RAW264.7 cells were seeded in 96 well plates at 5×10^3^ cells per well and were incubated in complete DMEM at 37°C, 5 % CO_2_ for 20–24 h. Cells were then stimulated with LPS (100 ng/mL) and simultaneously treated with 1, 5, 10, 20, 50, and 100 μM of DJ-X-013 and incubated at 37°C. After 24 h, the medium was replaced by fresh medium. 3-[4,5-dimethylthiazol-2-yl]-2,5 diphenyl tetrazolium bromide (MTT) reagent (Cat. no. M6494, Invitrogen, Carlsbad, CA, USA) [dissolved in phosphate-buffered saline (PBS)] was added in each well with final concentration of 0.5 mg/mL, and the plate was incubated in the dark at 37°C for 4 h. The medium containing MTT was carefully removed, the deposited formazan crystals were completely dissolved in dimethyl sulfoxide (DMSO) by incubating the plate in the dark at room temperature (RT) for 15 min., in shaking condition and the absorbance of each well was measured at a wavelength of 570 nm in a plate reader (CYTATION 5 imaging reader, Agilent BioTek, Santa Clara, CA). In this assay, the blank wells received the same treatment but without cells, while cells treated with only LPS served as controls for the evaluation of those treated with both LPS and DJ-X-013. The percentage of cell viability was calculated using the equation below.


$$Cell\;viability\;(\%)=\left[\left(A_{570}\right.\right.\;of\;Treated\;cells\;-\;Blank)/\left(A_{570}\right.\;of\;Untreated\;cells\;-\;Blank)]\;\times 100.$$

### 2.5. Phase-contrast imaging and morphometric analysis

Cultured RAW264.7 cell morphology of control, LPS, 10 μM, and 50 μM of DJ-X-013 treated cells were documented using an inverted phase contrast microscope (objective: 40×, final magnification 400×; AMG EVOS FL Life Technologies, Carlsbad, CA, USA). Morphometric analysis was performed on 40× images using ImageJ software (NIH). Ten representative images were selected from three experiments for each group and 5–8 cells per image were annotated using ImageJ software (NIH), for a total of 75 cells were analyzed per group. Each cell was assessed for six parameters, i.e., cell area, perimeter, major axis length, minor axis length, aspect ratio (major axis / minor axis), and circularity [4π x (Area / Perimeter^2^)].

### 2.6. Scratch or wound healing assay

Cell mobility was assessed using a scratch or wound healing assay. RAW264.7 macrophages were seeded in a 24-well plate, and incubated for 24 h, then the cell monolayer was scrapped in a straight line to create a scratch with a p200 pipet tip. Debris and detached cells were removed by washing with DMEM medium and an initial (0 h) image was photographed using an inverted phase contrast microscope (10× objective; AMG EVOS FL, Life Technologies). The cells were immediately treated with LPS and DJ-X-013, incubated at 37°C, and imaged in the same location at 4 h, 8 h, 24 h, and 28 h after treatment. The width of the wound was measured at each time point using ImageJ software (NIH) and the cell migration rate was calculated using the following equation:

$$R_{M}=\frac{W_{i}-W_{f}}{t}$$

where $R_{M}$ is the rate of cell migration (μm/h), $W_{i}$ is the initial wound width at 0 h (μm), $W_{f}$ is the final wound width (μm) at each time point, and t is the duration time (h).

### 2.7. Immunofluorescence (IF) staining and confocal imaging

After 24 h of treatment with LPS in the absence or presence of DJ-X-013 treatment as described above, RAW264.7 cells were fixed in 4 % paraformaldehyde (Cat. no. J19943-K2, Thermo Fisher Scientific, Waltham, MA, USA) and permeabilized with 0.1 % Triton X-100. Cells were blocked with 10 % goat serum in 1 % bovine serum albumin (BSA) and then incubated with rabbit primary antibody against mouse α-tubulin (Cat. no. A11126, Invitrogen) overnight at 4°C. After washing with PBS, the cells were subsequently incubated with Alexa fluor 488-conjugated goat anti-rabbit secondary antibody (Cat. no. A-11001, Invitrogen) at RT for 1 h. The cells were also stained with Texas red × phalloidin (Cat. no. T7471, Invitrogen) and counterstained with 4,6-diamidino-2-phenylindole (DAPI) (Sigma-Aldrich, St. Louis, MO, USA). Unbound reagents and antibodies were removed by washing with PBS at every step. Stained cells were mounted with ProLong^™^ Diamond Antifade mounting media (Cat. no. P36965, Invitrogen), allowed to dry, and microphotographs were grabbed using a Zeiss 710 confocal microscope (objective: 40×) in the institutional core facility.

### 2.8. Multiplex assays

We used a Milliplex MAP Multi-Pathway Total Magnetic Bead 9-plex Cell Signaling Multiplex Assay kit (Cat. no. 48–681MAG, Millipore Sigma, USA) to determine the total amounts of the following proteins in lysates of RAW264.7 cells treated with LPS in the absence or presence of DJ-X-013: cAMP response element-binding protein (CREB), p38, extracellular signal-regulated kinase/mitogen-activated protein kinase 1/2 (ERK/MAP1/2), STAT5, STAT3, NF-κB, RAC-α serine/threonine-protein kinase (AKT1), c-Jun N-terminal kinase (JNK), and ribosomal protein S6 kinase B1 (p70-S6k), as directed by the manufacturer.

We also used a Milliplex MAP Mouse Cytokine/Chemokine Magnetic Bead Panel Premixed 25 Plex Immunology Multiplex Assay kit (Cat. no. MCYTOMAG-70 K-PMX, Millipore Sigma) to analyze serum samples from mice with experimental colitis (described below). Briefly, each well of the assay plate was wetted with buffer and 25 μl beads were added to each. The blank well received 25 μl of assay buffer, while each sample well received a serum and the plate was shaken in the dark at 4°C overnight. The next day, each well was washed twice, 25 μl detection antibody was added to each well, and the plate was shaken in the dark at RT for 1 h. 25 μl streptavidin-phycoerythrin (PE) was added to each well, the plate was shaken in the dark at RT for 15 min., then amplification buffer was added and the plate was incubated as above for an additional 15 min. After the removal of streptavidin PE/amplification buffer, beads were resuspended in 150 μl assay buffer and analyzed using a Luminex^™^ System (Austin, TX) and software from Bio-Rad (Hercules, CA). The results were expressed as either mean fluorescence intensity (MFI) or picograms per milliliter (pg/mL).

### 2.9. Animal experiments

All animal experiments were performed under protocol no. 23–0450 approved by the University of Tennessee Health Science Center (UTHSC) Institutional Animal Care and Use Committee (IACUC). Wild-type (WT) C57BL/6 female mice (8 weeks old) were purchased from Jackson Laboratories (Bar Harbor, ME, USA) and housed in a specific pathogen-free animal facility at UTHSC, Memphis with normal 12/12 h light/dark cycles. The mice were housed for a week for acclimatization to the animal facility before starting the experiment. Following one week of acclimatization, the mice were randomly divided into three experimental groups, each containing five mice (n=5/group): control, DSS alone, and DSS+DJ-X-013 (designated DJ-X-013 throughout the experiment). DSS was used to induce experimental colitis in the mice, as described [27]. Briefly, on the day the experiment started (day 0), the DSS and DJ-X-013 groups were provided DSS (Molecular weight: 36–50 kDa; Cat. no. 160110, MP Biomedicals, Santa Ana, CA, USA) at 3.5 % in the drinking water, which was changed at two-day intervals, and the treatment was continued up to day 6 for a total of 7 days. Thereafter, the DSS water was replaced with plain drinking water. In a preliminary dose-response experiment using DJ-X-013 at doses of 20, 40, and 80 mg/kg body weight, the 20 mg/kg dose was found to effectively reduce colitis symptoms relative to the higher doses (Supplementary Fig. 1). Therefore, a 20 mg/kg dose of DJ-X-013 was used for this study. Mice in the DJ-X-013 group were administered daily doses of 20 mg/kg DJ-X-013 (dissolved in a standardized ratio of DMSO, ethanol, and PBS) in 100 μl volume via oral gavage on day 1 and continuing to day 7. During days 1–7, the control and DSS groups were administered 100 μl vehicle (same ratio of solvents). The mice were monitored daily for body weight, behavior, and clinical symptoms of colitis (diarrhea, stool consistency, and blood in fecal matter). The mice were euthanized at the experimental endpoint on day 8 and spleen, mesenteric lymph nodes (MLNs), colon tissues, and whole blood (from which serum was collected by centrifugation) were collected for further downstream experiments. The experiment was repeated three times to achieve statistical significance.

### 2.10. Single-cell isolation from spleen and mesenteric lymph nodes (MLNs)

After removing the fat bodies, the spleen and MLNs were collected in ice-cold complete Roswell Park Memorial Institute Medium 1640 (RPMI 1640; Cat. no. 10–041-CV, Corning). Tissues were homogenized for 30–45 s. in a Seward^™^ Stomacher^™^ 80 lab blender (Fisher Scientific), the cell suspension was passed through a 70 μm filter, and the cells were collected by centrifugation (300 x g) at 4°C for 10 min. MLNs cells were resuspended in RPMI medium and the total cell number and percentage of live cells were estimated in the Invitrogen cell counter after staining with trypan blue. Spleen cells were incubated with red blood corpuscle lysis buffer (Cat. no. 00–4333–57, Invitrogen) for 3–4 min., resuspended in complete RPMI, and the cells were collected by centrifugation (300 x g) at 4°C for 10 min. The cell pellet was resuspended in complete RPMI, and the cells were passed through a 70 μm filter and counted as described above.

### 2.11. Lymphocyte isolation from colon lamina propria (LP)

We isolated total lymphocytes from the colon lamina propria using a mouse lamina propria dissociation kit (Cat. no. 130–097–410, Miltenyi Biotec, Auburn, CA, USA) according to the manufacturer’s protocol. Briefly, the colons were collected and cleaned in Hank’s balanced salt solution (HBSS). Colons were cut longitudinally, then transversely into small pieces (approx. 0.5 cm in length), placed 50 mL falcon tube, and incubated at 37°C in first pre-digestion solution (2×20 min.), then HBSS (20 min.), then digestion solution containing enzymes A, D, and R (30 min.) in a MACS^™^ C tube (Cat. no. 130–093–237, Miltenyi Biotec), in a MACSmix tube rotator with continuous rotation. The samples were transferred to a gentle MACS^™^ Dissociator (Cat. no. 130–093–235, Miltenyi Biotec) and dissociated using the gentle MACS program “m_intestine_01”. After a brief centrifugation to collect the cells, the cell pellet was resuspended in PB buffer (PBS with 0.5 % bovine serum albumin), and the cells were passed through a 100 μm filter. The cells were collected by centrifugation, resuspended in a complete RPMI medium, and counted in a counting chamber after trypan blue staining before further analysis.

### 2.12. Flow cytometry analysis

Cultured RAW 264.7 cells or cells isolated from mouse spleen, MLNs, or colon LP were washed and resuspended in ice-cold flow cytometry staining buffer (FACS buffer; PBS with 1 % FBS). For surface staining, the manufacturer’s recommended concentration of the appropriate antibodies was added to the cells and incubated in the dark at 4°C for 40 min. with occasional shaking. Details of flow antibodies are described in Table 1; all fluorescence-conjugated antibodies and buffers used for intracellular staining were purchased from Biolegend (San Diego, CA). For intracellular staining, the cells were fixed and permeabilized sequentially in fixation/permeabilization buffer, then permeabilization buffer, the appropriate antibodies were added, and the cells were incubated at RT for 30 min. After removal of unbound antibodies by washing, cells were resuspended in 300 μl FACS buffer and analyzed in a Novocyte flow cytometer (Agilent Technologies, Santa Clara, CA).

### 2.13. RNA isolation and reverse transcription-quantitative polymerase chain reaction (RT-qPCR)

Total RNA was extracted from RAW 264.7 cells and colon tissue using an RNeasy Mini kit (Cat. no. 74104, QIAGEN, Germantown, MD, USA) following the manufacturer’s protocol. The concentration and purity of the RNA were determined using a Nanodrop spectrophotometer (Fisher Scientific). For each sample, 250 ng of extracted RNA was used as a template by reverse transcriptase for cDNA synthesis using an iScript cDNA synthesis kit (Cat. no. 1708891, Bio-Rad) according to the manufacturer’s protocol. This cDNA was subsequently used as a template for qPCR using iTaq Universal SYBR Green Supermix (Cat. no. 1725121, Bio-Rad). All primers were purchased from Integrated DNA Technologies (IDT; Coralville, IA, USA) and the details of the primer sequences used to amplify the selected genes are provided in Table 2.

### 2.14. Immunoblot (IB) analysis

RAW 264.7 cells and colon tissue were lysed with radioimmunoprecipitation assay (RIPA) buffer (Cat. no. J63306, Alfa Aesar, Ward Hill, MA, USA) supplemented with Halt^™^ protease and phosphatase inhibitor cocktail (Cat. no. 78442, Thermo Scientific). Complete cell lysis was achieved by vigorous pipetting, tissue homogenization, sonication, and incubation on ice for 30 min. Cell debris was removed by centrifugation at 16,000 × g at 4°C for 20 min. and the total protein concentration of the supernatant was estimated using a Pierce^™^ BCA Protein Assay Kit (Cat. no. 23225, Thermo Fisher Scientific). Equal amounts (20 μg) of protein for each sample were loaded on a 10 % gel and separated through sodium dodecyl sulfate-polyacrylamide gel electrophoresis (SDS-PAGE). The gel was casted using a TGX^™^ FastCast^™^ Acrylamide Starter Kit (Cat. no. 1610172, Bio-Rad) and seperated proteins were transferred to polyvinylidene fluoride (PVDF) membranes (Cat. no. 1620177, Bio-Rad) using a Trans-Blot Turbo Transfer System (Cat. no. 1704150, Bio-Rad). The membrane was blocked with blocking buffer (Cat. no. 927–60001, LI-COR Biosciences, Lincoln, NE, USA) at RT for 2 h and incubated with the following primary antibodies at 4°C overnight with shaking: NF-κB p65 (1:1000; Cat. no. 8242 T, Cell Signaling Technology, Danvers, MA, USA) or NF-κB p100–50 1:1000; Cat. no. 4882S, Cell Signaling Technology). Antibody against the housekeeping protein β-actin (1:5000; Cat. no. 926–42212, LI-COR) was used as a loading control. Unbound primary antibodies were removed by washing with Tris-buffered saline (TBS) containing Tween^®^ 20 (polyethylene glycol sorbitan monolaurate) and the membranes were incubated in the dark at RT for 1 h with the following fluorophore-conjugated secondary antibodies: goat anti-rabbit IgG (1:5000; Cat. no. 926–32211, LI-COR Biosciences) and goat anti-mouse IgG (1:5000; Cat. no. 926–68070, LI-COR Biosciences). After washing to remove excess secondary antibodies, the membrane was visualized and imaged using a LICOR Odyssey^®^ DLX imaging system (LI-COR Biosciences). Densitometric analysis was performed using ImageJ software (NIH).

### 2.15. Hematoxylin and eosin (H&E) staining, imaging and measurement of colon layers width

Mice distal colons were fixed using 4 % paraformaldehyde (Cat. no. J19943-K2, Thermo Scientific) for 24 h and embedded in paraffin. Fixed tissues were cut into 5 μm sections, deparaffinized, stained with hematoxylin and eosin, and examined using a bright field microscope (Model no. BX43, Olympus Life Science Solutions/Evident Scientific, Tokyo, Japan) in different magnifications. The inflammation score was estimated based on the severity of epithelial cell disruption, loss of crypt structure, loss of goblet cells, and the degree of inflammatory cell infiltration. The inflammatory state of each colon was characterized and scored as follows: having no change when compared with tissue samples from control mice (score = 0); having a few mononuclear cell infiltrates (score = 1); having minimal mononuclear cells (score = 2); having a medium level of infiltration (score = 3); or exhibiting loss of epithelial cells with heavy cellular infiltrates in the sub-mucosa (score = 4). Furthermore, colon epithelial folding length and sub-mucosal width were semi-quantitatively estimated from H&E images using ImageJ software (NIH).

### 2.16. Statistical analysis

All data are shown as mean values ± standard error of the mean (SEM). Statistical analysis was performed using one-way analysis of variance (ANOVA) followed by either Tukey’s or Dunnett’s multiple comparison tests (as indicated in the figure legends) to determine the significance level. A p-value of 0.05 was considered the level of significance in all analyses (ns [not significant] p > 0.05, * p < 0.05, ** p < 0.01, *** p < 0.001 and **** p < 0.0001). Graphical representations were generated using GraphPad Prism software (GraphPad Software, Boston, MA).

## 3. Results

### 3.1. DJ-X-013 exhibited high gastrointestinal (GI) absorption, exclusion by the blood-brain barrier (BBB), and optimized physical properties

The non-steroidal thiazole-based compound DJ-X-013 was designed as a small candidate molecule anti-inflammatory drug. Rapid progress in drug discovery relies on the early assessment of a candidate molecule’s absorption, distribution, metabolism, and excretion (ADME) properties. This process can be greatly aided by the use of computer prediction programs such as SwissADME [39], which we used as an initial approach to evaluate these properties for candidate drug DJ-X-013. The ADME properties of DJ-X-013 are shown in Figs. 1A and 1B. The BOILED-Egg model (Fig. 1A) allows simultaneous prediction of passive gastrointestinal (GI) absorption and brain penetration, with the BBB permeation region, represented as a round, yellow yolk-like sphere, the human intestinal absorption (HIA) region as a white oval, and the low absorption and limited brain permeation region as a grey rectangle [39]. In this image (Fig. 1A), the queried molecule DJ-X-013 is shown within the HIA as a red hollow sphere whose color and location indicate that

The pink shaded area at right of Fig. 1B represents the physicochemical space that is suitable for oral bioavailability, while DJ-X-013 is shown as a red line, calculated from its physical properties, which were assessed based on its predicted lipophilicity (LIPO), size (SIZE), polarity (POLAR), insolubility (INSOLU), insaturation (INSATU), and flexibility (FLEX). The key physicochemical, pharmaceutical, and drug-likeness properties of DJ-X-013 are summarized in Table 3. DJ-X-013 was predicted to have high GI absorption, no penetration of the BBB, and high bioavailability. Traditionally, small-molecule drugs with therapeutic activity comply with Lipinski’s Rules of Five [41], i.e., having molecular mass less than 500 Da, fewer than five hydrogen-bond donors, fewer than ten hydrogen-bond acceptors, and an octanol-water partition coefficient (log *P*_o/w_) less than 5. By these criteria (Table 3), DJ-X-013 was predicted to have therapeutic activity. Finally, we used two different techniques, methods A and B, to synthesize DJ-X-013, as detailed in Materials and Methods and summarized in Fig. 1C. The final product of both synthesis methods (compound DJ-X-013) shared the same physicochemical properties and was tested for its biological activity.

### 3.2. DJ-X-013 altered the viability of cultured RAW264.7 macrophages

To determine the effect of DJ-X-013 on cell viability (cytotoxicity), we treated LPS-activated RAW264.7 macrophages with DJ-X-013 over a range of different concentrations for 24 h and used MTT assay to examine their percent viability relative to LPS alone (100 ng/mL). When DJ-X-013 was used at a concentration range of 5–20 μM, cells retained viability of 79.3 ± 3.8 % to 75.3 ± 0.5 %, whereas at a concentration of 100 μM, only 22.4 ± 2.1 % of cells remained alive after 24 h (Fig. 1D). We used these results in a linear equation (y=mx+c, where x and y are variables, m is the slope, and c is the y-intercept) to calculate LC_50_ of 59.5 μM, indicating that treatment of these cells with DJ-X-013 induced 50 % cell death at a concentration of approximately 60 μM. Based on these results, we selected concentrations of 10 μM and 50 μM doses for our downstream *in vitro* experiments.

### 3.3. DJ-X-013 restored the morphology of RAW 264.7 macrophages after LPS stimulation

Cell morphology or phenotype is the basic outcome of cellular function. RAW 264.7 macrophages without treatment were used as a control in all downstream *in vitro* experiments including morphometric analysis by phase contrast microscopy (Fig. 1E); these control cells exhibited a morphology that was mostly round. On treatment with LPS alone, these cells exhibited two distinct morphologies: round (R) and elongated (E), the latter of which are indicated in the figure by red arrows (Fig. 1E). Interestingly, after treatment of these cells with LPS and 10 μM or 50 μM DJ-X-013, the elongated cells were diminished from the population and the round morphology predominated (Fig. 1E).

To further evaluate the cell morphology under these conditions, we performed cell morphometric analysis using ImageJ software (NIH). Six morphometric parameters were analyzed, including area, perimeter, major axis, minor axis, aspect ratio, and circularity (Fig. 1F). While the area and perimeter are descriptors of cell size, the major axis, minor axis, aspect ratio, and circularity parameters allowed us to examine the cell shape as either round or elongated. For LPS-treated cells, the cellular area was significantly enlarged in both R and E morphologies relative to that of control cells and those of the LPS (E) morphology had a larger area than cells treated with LPS plus either 10 μM or 50 μM DJ-X-013 (Fig. 1F). Interestingly, the cells with the LPS (R) morphology exhibited a smaller area than the cells treated with LPS and 10 μM or 50 μM DJ-X-013. The perimeter and major axis parameters exhibited a similar trend except when comparing cells of the LPS (R) morphology to cells treated with LPS and 10 μM DJ-X-013.

Furthermore, the area, perimeter, and major axis were all highest for LPS (E) cells, although the minor axis of these cells was highly subsided (as expected for the elongated cell morphology). The minor axis increased gradually after treatment with LPS plus either concentration of DJ-X-013, relative to the minor axis of LPS (E) cells. The alteration of the major and minor axis was further reflected in the aspect ratio, which was highest for cells of the LPS (E) morphology, while these cells exhibited the lowest circularity. In contrast, there were no significant differences in aspect ratio and circularity when comparing cells of the LPS (R) morphology to those of cells treated with LPS plus 10 μM or 50 μM DJ-X-013.

Considering that the aspect ratio and circularity values of a circle are both equal to 1, an aspect ratio > 1 indicates a higher degree of elongation, while a circularity value < 1 indicates a higher degree of protrusions generation [42]. Thus, the elevated aspect ratio and lower circularity values of LPS (E) cells emphasized their morphological differences. Furthermore, the distribution of data points for every parameter of LPS (E) cells was very scattered relative to the other cells, suggesting that their morphology was very dynamic. Thus, the results of cellular morphometric analysis after phase contrast microscopy suggested that LPS may induce polarization of cultured RAW264.7 macrophages to an elongated morphology with more protrusions and that the addition of DJ-X-013 after LPS-induction either inhibits the cellular elongation-induced by LPS or reverts the elongated cell morphology to the round morphology despite LPS stimulation. Next, we examined the functional inhibition of LPS-treated RAW 264.7 cells by DJ-X-013 using a scratch or wound healing assay to examine their migration ability.

### 3.4. DJ-X-013 attenuated LPS-stimulated RAW 264.7 cell migration

**Fig. 2.**
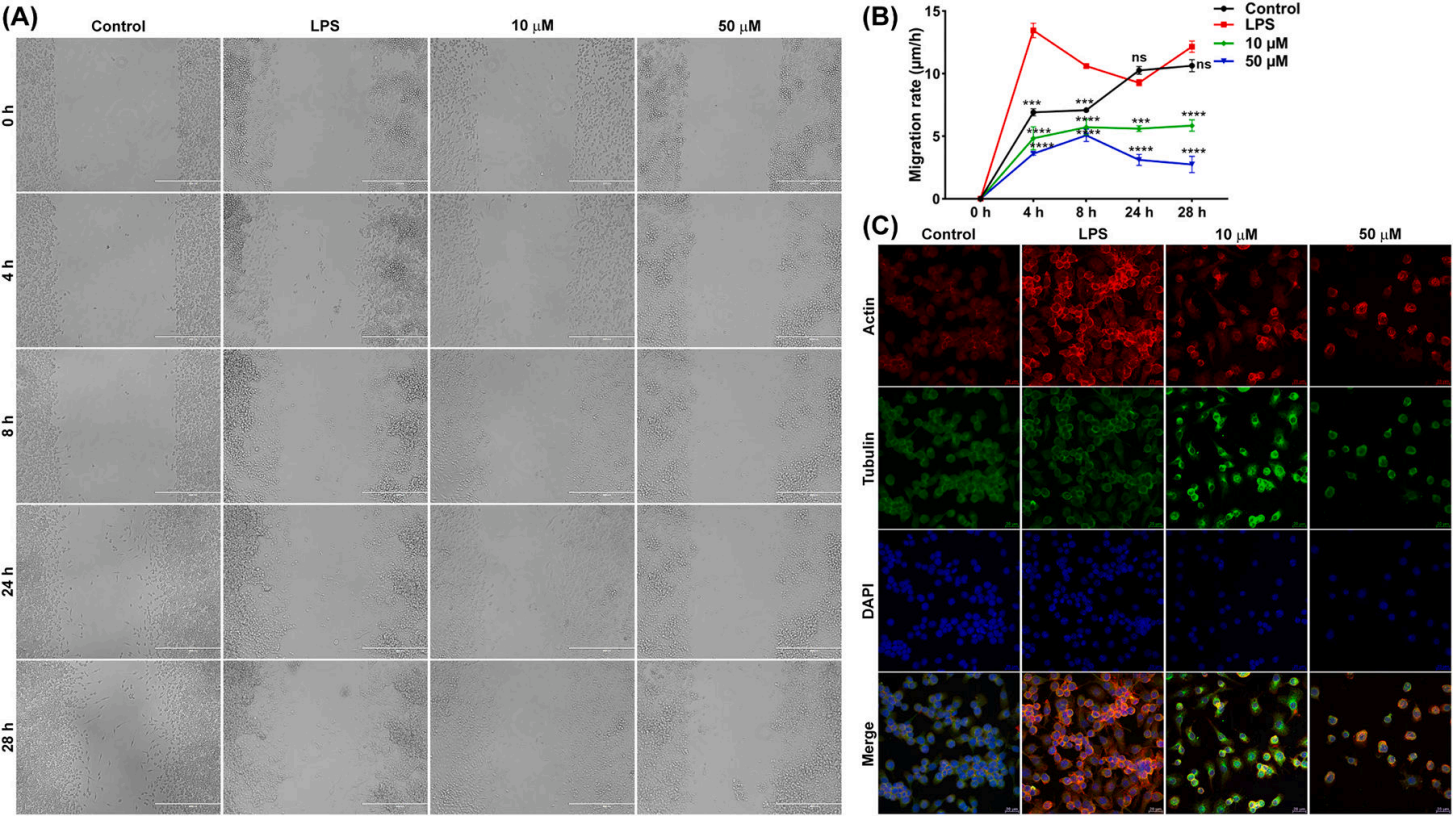
DJ-X-013 attenuated *in vitro* LPS-stimulated RAW 264.7 cell migration by regulating the expression of cytoskeletal proteins. (A) Representative phase contrast images show the closure of a scratch wound in a monolayer of LPS-stimulated RAW 264.7 macrophages in the presence or absence of DJ-X-013 over time, following incubation of 0, 4, 8, 24, and 28 h; scale bar 400 μm. (B) Plot showing that DJ-X-013 reduces cell migration rate. Statistical analysis was performed using one-way ANOVA followed by Dunnett’s post hoc test; n = 3. Data are presented as mean values ± SEM, ns p > 0.05; ***p < 0.001; ****p < 0.0001. (C) Representative immunofluorescence confocal microscopic images depict the alteration of actin and tubulin after treatment of LPS-stimulated RAW 264.7 macrophages with 10 or 50 μM DJ-X-013. Scale bar 20 μm; detected proteins (color): F-actin (red), tubulin (green), and DAPI for nucleus (blue). These results suggest that DJ-X-013 impedes the migration of macrophages and might modulate the polymerization or depolymerization of cytoskeletal proteins.

The scratch or wound healing assay monitors the potential of a cell to migrate and we have used it to calculate the cell migration speed of LPS-stimulated RAW 264.7 cells in the presence or absence of DJ-X-013 by measuring the width of the scratch at different temporal points (Fig. 2A, 2B). The migration rate was highest after 4 h of LPS treatment, relative to that of the control. In contrast, cells stimulated with LPS and treated with 10 μM or 50 μM DJ-X-013 maintained a slow migration speed from 4 to 28 h, relative to that of control cells and cells treated with LPS. In general, LPS serves to stimulate the migration of macrophages to infected and inflamed tissue. Here, LPS treatment activated RAW264.7 macrophages to migrate to fill the scratch or wound site, whereas this migration was slowed on treatment with DJ-X-013, suggesting that this compound impaired the LPS-induced cell migration. To investigate this phenomenon in more detail, we examined the expression of cytoskeleton proteins required for RAW264.7 cell motility in the absence and presence of DJ-X-013.

### 3.5. DJ-X-013 treatment of LPS-stimulated RAW 264.7 cells altered the expression of cytoskeletal proteins

Cell morphology and migration are regulated by crosstalk between cytoskeletal proteins, specifically actin filaments, tubulin microtubules, and intermediate filaments. Thus, we examined the effect of 10 or 50 μM DJ-X-013 treatment of LPS-stimulated RAW 264.7 macrophages on actin and tubulin proteins using immunofluorescence (IF) confocal microscopy. In the LPS group, actin became polymerized, while in the LPS+10 μM DJ-X-013 group, tubulin became polymerized, resulting in bright fluorescence signals (Fig. 2C). Cell migration involves dynamic polymerization and depolymerization of actin and tubulin, while inhibition of any of these processes causes defects in cell migration, cell shape, and intracellular transport. Thus, these IF results suggest that DJ-X-013 may modulate actin depolymerization, tubulin polymerization, or both. We next examined the effect of DJ-X-013 treatment on other cellular activities, including gene expression and protein synthesis.

### 3.6. DJ-X-013 impeded the expression of inflammatory markers in LPS-stimulated RAW 264.7 cells

**Fig. 3.**
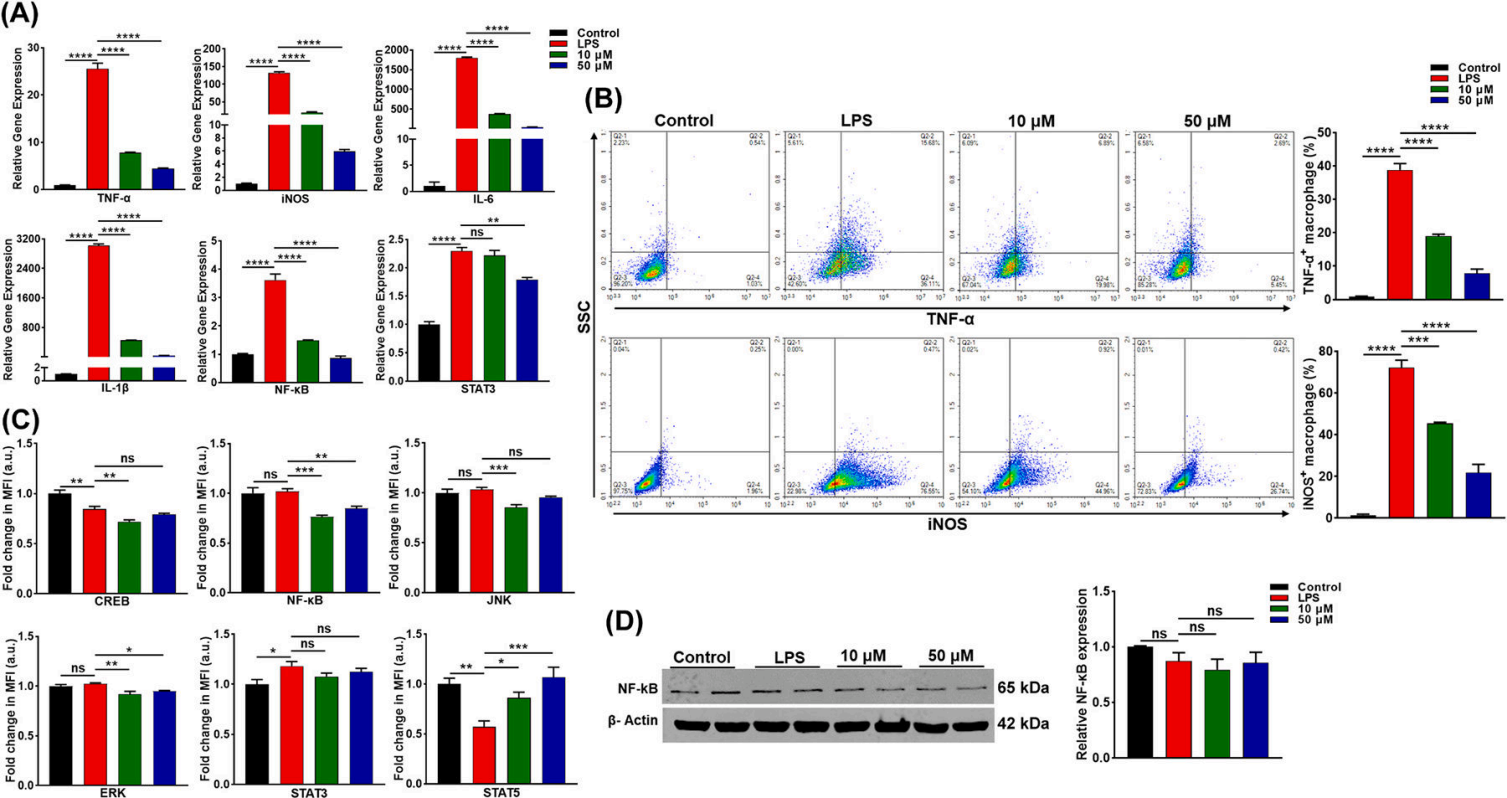
Treatment of LPS-stimulated RAW 264.7 cells *in vitro* with DJ-X-013 diminishes the expression of inflammatory markers. (A) In LPS-stimulated RAW 264.7 cells treated in the absence or presence of 10 or 50 μM DJ-X-013, the expression of the mRNAs encoding pro-inflammatory markers TNF-α, iNOS, IL-6, IL-1β, STAT3, and NF-κB was analyzed using reverse transcription-quantitative polymerase chain reaction (RT-qPCR). While LPS alone stimulated the expression of these inflammatory genes, it was inhibited by DJ-X-013. (B) After a similar treatment of RAW 264.7 cells, flow cytometry was used to examine the expression of TNF-α and iNOS proteins, lower right quadrants of the top and bottom rows show the percentage of TNF-α+ and iNOS+ cells, respectively. The representative density plot and related bar graph with analysis from repeated experiments are shown. This experiment shows that DJ-X-013 treatment reduced TNF-α+ and iNOS+ expression in LPS-stimulated RAW 264.7 cells. (C) Multiplex assay analysis of CREB, ERK, JNK, STAT3, STAT5, and NF-κB signaling after similar treatment. The unit mean fluorescence intensity (MFI) was directly proportional to the concentration. (D) After similar treatment, cells were lysed, total protein extracted and the NF-κB protein expression was analyzed by immunoblot. In totality, DJ-X-013 reduced the expression of mRNAs and proteins encoded by inflammation-associated genes. Statistical analysis was performed by one-way ANOVA followed by Dunnett’s post hoc test for (A) n = 3, (B) n = 3, (C) n = 4–5, and (D) n = 3. Data are presented as mean values ± SEM, ns p > 0.05; *p < 0.05; **p < 0.01; ***p < 0.001; ****p < 0.0001.

We examined the effect of DJ-X-013 on the expression of inflammatory genes and proteins in LPS-stimulated RAW 264.7 cells (Fig. 3). In RAW 264.7 cells, LPS induces the expression of various inflammatory markers, including TNF-α, inducible nitric oxide synthase (iNOS), interleukin 6 (IL-6), and IL-1β, which in turn stimulate inflammatory signaling pathways such as NF-κB and cJun N-terminal kinase (JNK)/STAT3. We explored the modulatory role of DJ-X-013 on inflammatory marker expression in LPS-stimulated RAW 264.7 cells using RT-qPCR (Fig. 3A), flow cytometry (Fig. 3B), multiplex assay (Fig. 3C) and immunoblot analysis (Fig. 3D).

LPS treatment of RAW 264.7 stimulated expression of TNF-α, iNOS, IL-6, IL-1β, NF-κB, and STAT3 relative to the control, while treatment with LPS together with 10 μM or 50 μM DJ-X-013 significantly reduced the expression of these genes (Fig. 3A). Interestingly, treatment of the cells with 50 μM DJ-X-013 diminished the expression of STAT3 expression relative to that in the LPS group. Analysis of TNF-α and iNOS expression by flow cytometry showed that DJ-X-013 treatment of LPS-stimulated cells reduced the expression of both proteins (Fig. 3B). Further, the multiplex bead array assay showed that treatment with 10 μM DJ-X-013 strongly decreased the expression of CREB, NF-κB, JNK, and ERK relative to LPS alone (Fig. 3C). Oppositely, DJ-X-013 enhanced STAT5 expression. The expression of p38, p70S6k, and Akt was not altered prominently after LPS and DJ-X-013 treatment (Supplementary Fig. 1). Finally when we examined NF-κB expression using an immunoblot assay, we observed a slight decrease in its expression in the presence of LPS showing a diminishing trend (Fig. 3D). Overall, DJ-X-013 effectively reduced the expression of NF-κB, inflammatory markers, and common signaling pathways in RAW 264.7 macrophages after LPS stimulation.

### 3.7. DJ-X-013 reduced colon inflammatory parameters associated with colitis

**Fig. 4.**
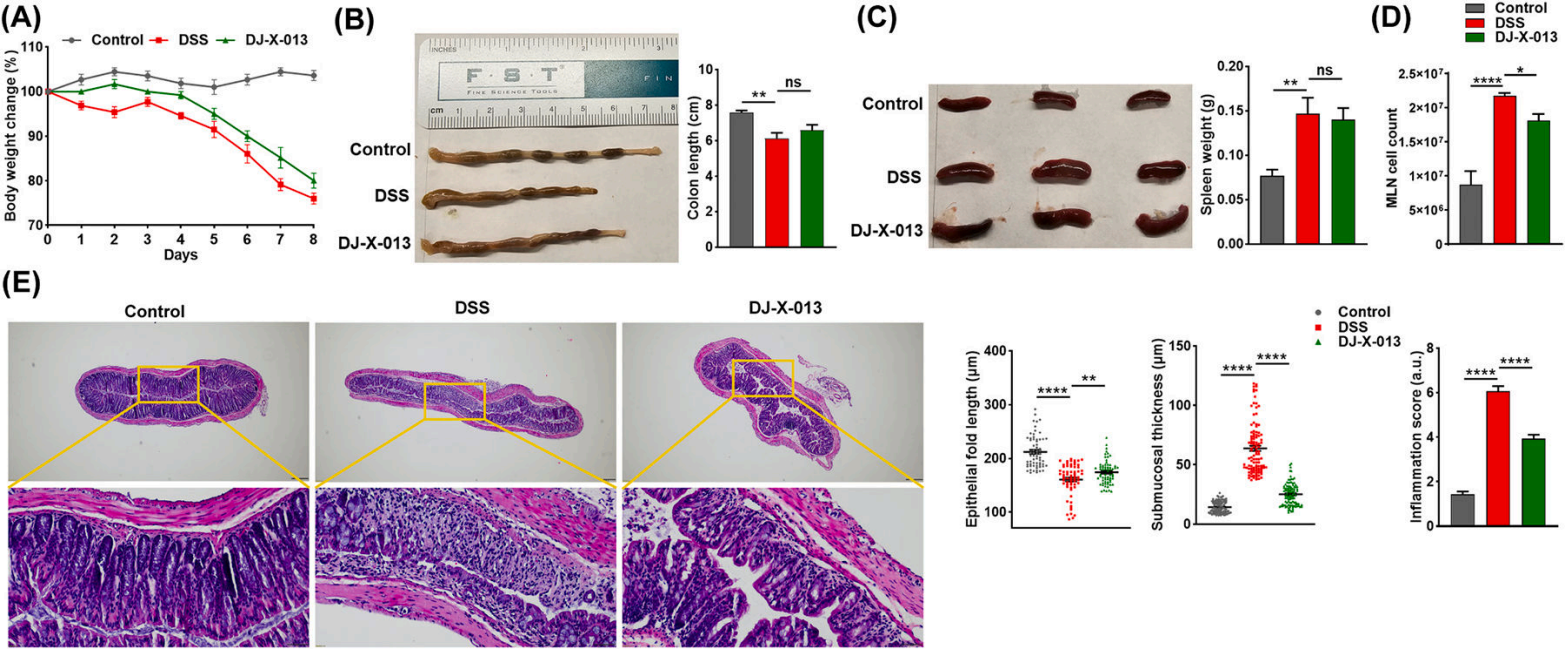
DJ-X-013 treatment ameliorated DSS-induced colitis in mice. Change in body weight and colon length after DJ-X-013 treatment in DSS-induced colitis. C57BL/6 mice received daily treatment in drinking water for 7 days as follows: control mice received plain water + vehicle (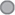), DSS-treated mice received 3.5 % DSS + vehicle (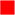), and DSS + DJ-X-013 mice received 3.5 % DSS + 20 mg/kg body weight DJ-X-013 (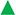). (A) The body weight of each mouse was recorded every day and the change from the initial body weight was expressed as a percentage change in body weight (n = 5). (B) Macroscopic view of colon length and related plot (C) Macroscopic view of spleen size and related plot of spleen weight. (D) MLNs were isolated from each mouse and MLN cells were isolated and counted; shown is the count per mouse. (E) Histological analysis of H&E-stained colon tissue from each experimental group is shown in representative microphotographs. Scale bars 200 μm in upper panels and 50 μm in lower panels, respectively. The epithelial fold length and sub-mucosal width or thickness were measured from histological images using ImageJ software (NIH) and the inflammation score was determined based on epithelial damage, loss of goblet cells, crypt disruption, and infiltration of inflammatory cells. Statistical analysis was performed using one-way ANOVA followed by Dunnett’s post hoc test in (B) n = 5, (C) n = 6, (D) n = 5–8, and (E) n = 65 for epithelial fold length, n = 105 for submucosal thickness and n = 16 for inflammation score. Data are presented as mean values ± SEM, ns p > 0.05; *p < 0.05; **p < 0.01; ****p < 0.0001.

We also examined the effect of DJ-X-013 on body weight and colon length *in vivo* in a DSS-induced colitis model in mice. Since we observed no drastic change in body weight for the entire experimental period in naïve mice, we compared the body weight of mice receiving DSS with those receiving DSS + DJ-X-013; the change from the initial body weight was expressed in terms of percentage of body weight change. While the body weight in both groups progressively declined over time, mice that received DSS alone developed colitis, as shown by a greater decrease in body weight and mice treated with DSS + DJ-X-013 exhibited an improvement in body weight as compared to the DSS group (Fig. 4A & Supplementary Fig. 2). After the experimental endpoint, the mice were euthanized and colons were isolated and measured. We observed that the mean colon length of mice with DSS-induced colitis was slightly shorter than that of the colons of DJ-X-013 treated mice, although the difference was not statistically significant (Fig. 4B). This data suggests that administration of DJ-X-013 may have a protective role in maintaining colon length. To obtain a preliminary idea about splenomegaly and inflammation in these mice, we determined the spleen weight and MLNs cell number. The size and weight of the spleen increased in the DSS group, while spleen weight decreased marginally in mice treated with DSS and DJ-X-013 (Fig. 4C). The total number of MLNs cells significantly decreased in the DJ-X-013 group compared to mice treated with DSS alone (Fig. 4D).

We also evaluated colon pathology, including epithelial fold length, submucosal thickness, and inflammation score (Fig. 4E). The colon pathology in mice with DSS-induced colitis showed loss of colonocyte organization in the epithelium, a hypertrophied submucosal layer, and an increased inflammatory score (Fig. 4E), predominantly due to infiltration by immune cells. In contrast, mice treated with DSS and DJ-X-013 exhibited improved colon histology and a reduced inflammation score relative to the mice treated with DSS alone. The tissue parameters associated with colitis, which include epithelial fold length and submucosal thickness of the colon, were significantly improved following treatment with DJ-X-013 (Fig. 4E). Taken together, these outcomes suggest that DJ-X-013 treatment ameliorated colitis in DSS-induced mice by restoring body weight, spleen weight, colon length, and epithelial architecture and reducing inflammatory cell infiltration. The outcome that inflammatory cell infiltration might be altered by treatment with DJ-X-013 was followed up by flow cytometry analysis.

### 3.8. DJ-X-013 treatment inhibited the infiltration of TNF-α producing macrophages, activated monocytes, and neutrophils in the colon of DSS-induced mice

**Fig. 5.**
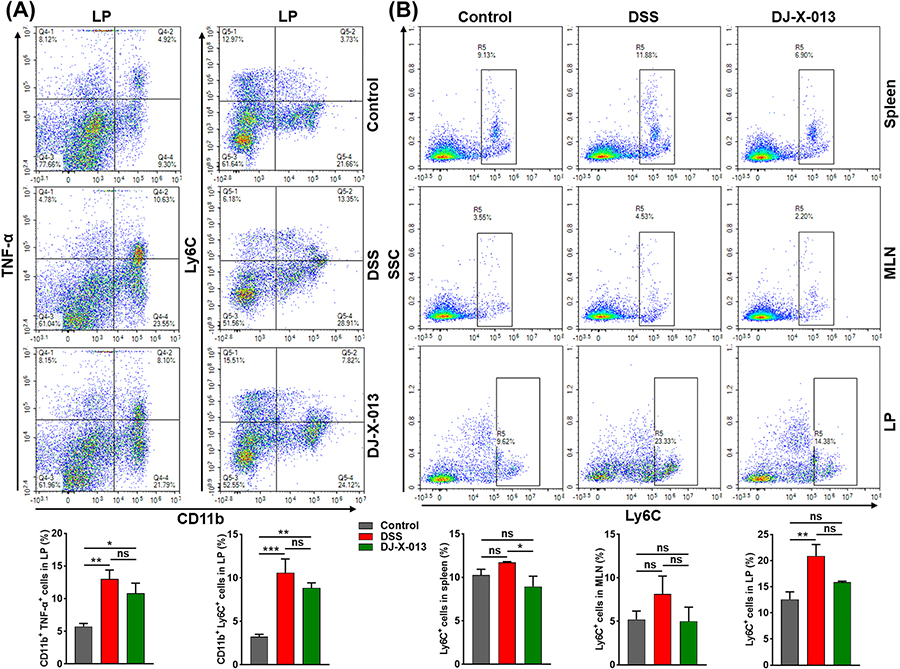
DJ-X-013 treatment of mice resolved DSS-induced colitis by inhibiting TNF-α-producing macrophages, activated monocytes, and neutrophils. Spleens, MLNs, and colon LP immune cells were isolated from the three groups of mice (control, DSS, DSS + DJ-X-013) on day 8 and stained with antibodies specific for CD11b^+^, TNF-α, and Ly6C. (A) The percentages of CD11b^+^ macrophages producing TNF-α (left column) and of activated monocytes (CD11b^+^ Ly6C^+^; right column) are shown in the upper right quadrants. (B) The percentages of neutrophils detected in the indicated organ are shown (Ly6C^+^ cells are boxed). The representative density plot and related bar graph with analysis from repeated experiments are shown. Statistical analysis was performed using one-way ANOVA followed by Tukey’s multiple comparison post hoc tests; n = 6. Data are presented as mean values ± SEM, ns p > 0.05; *p < 0.05; **p < 0.01; ***p < 0.001.

Macrophages express the pro-inflammatory cytokine TNF-α while neutrophils play a role in the development and maintenance of intestinal inflammation and occur in increased numbers during DSS-induced colitis [43]. Levels of TNF-α increase in IBD patients and blocking TNF-α production is a crucial aspect of current IBD therapeutics [44]. Therefore, we used flow cytometry to examine the frequency of TNF-α produced by mucosal macrophages and systemic neutrophils in DSS-induced colitis in the absence or presence of DJ-X-013. Importantly, we observed a reduction in the frequency of inflammatory cells i. e. CD11b^+^ macrophages producing TNF-α and CD11b^+^ Ly6C^+^ inflammatory monocytes in the colon LP after treatment with DSS and DJ-X-013 (Fig. 5A). In mice treated with DSS alone, the percentage of neutrophils increased both in systemic and colon lamina propria (LP), relative to that in control mice (Fig. 5B). In contrast, after treatment of mice with DSS and DJ-X-013, the frequency of neutrophils was reduced in the spleen, MLNs, and colon LP, relative to that observed in mice treated with DSS alone. Though, the statistical analysis showed no significant difference however, taken together these results’ trend suggests that treatment with DSS and DJ-X-013 attenuated the number of macrophages, monocytes, and neutrophils in the mouse colon and that DJ-X-013 may be effective in reducing colitis.

### 3.9. DJ-X-013 decreased the number of mucosal-activated T cells and revised NK/NKT cells

**Fig. 6.**
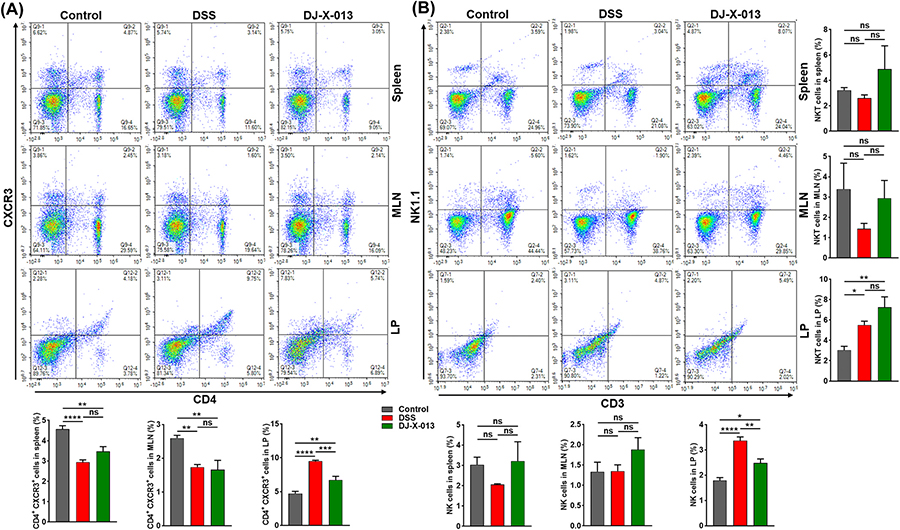
DJ-X-013 treatment of mice with DSS-induced colitis improved colitis symptoms by differentially modulating T cells and natural killer cells. Spleens, MLNs, and colon LP immune cells were isolated from the three groups of mice (control, DSS, DSS + DJ-X-013) on day 8 and stained with antibodies specific for CD3, CD4, CXCR3, and NK1.1. (A) The percentages of activated T cells (CD4^+^CXCR3^+^) are shown in the upper right quadrants. (B) The percentages of NK cells (CD3^−^ NK1.1; upper left quadrants) and NKT cells (CD3^+^ NK1.1; upper right quadrants) are shown. The representative density plot and related bar graph with analysis from repeated experiments are shown. Statistical analysis was performed using one-way ANOVA followed by Tukey’s multiple comparison post hoc tests; n = 6. Data are presented as mean values ± SEM, ns p > 0.05; *p < 0.05; **p < 0.01; ***p < 0.001; ****p < 0.0001.

We showed previously that inflammation in the mouse colon is primarily mediated by CD4^+^ T helper 1 lymphocytes (Th1 cells) [45,46]. Further, natural killer (NK) cells and natural killer T (NKT) cells participate in the regulation of the intestinal immune response [47], and in differentiating naive CD4^+^ T cells into Th1 cells [48]. Thus, we used flow cytometry analysis to explore the effect of DJ-X-013 treatment on populations of activated CD4^+^ T cells isolated from spleen, MLN, and colon LP of DSS + DJ-X-013 treated mice (Fig. 6A). While the frequency of activated T cells (CD4^+^CXCR3^+^) in the spleens and MLNs was not much altered after DJ-X-013 treatment, the percentage of activated T cells in the colon LP declined significantly in the DJ-X-013 group relative to the DSS alone group (Fig. 6A, upper right quadrants and related bar graphs). These findings suggest that treatment of mice with DJ-X-013 markedly reduces the frequency of CD4^+^CXCR3^+^ T cells in the colon LP, which may protect them against DSS-induced colitis.

We also examined the frequency of mucosal and systemic NK and NKT in mice treated with DSS in the absence or presence of DJ-X-013 treatment. The induction of colitis in the mice with DSS slightly decreased the percentage of NK (Fig. 6B, upper left quadrants) and NKT (Fig. 6B, upper right quadrants) cells in the spleen and MLNs, relative to the control mice. Treatment with DJ-X-013 increased the percentages of NK and NKT cells in the spleens and MLNs relative to DSS alone (Fig. 6B). However, in the colon LP, both NK and NKT cell populations increased surprisingly in mice treated with DSS alone, relative to the control (Fig. 6B, upper left and right quadrants), while treatment with DSS + DJ-X-013 was associated with a decreased percentage of NK cells and marginally elevated NKT cell infiltration in colon LP, relative to the percentages observed in mice treated with DSS alone. Taken together, results suggest that treatment with DJ-X-013 differentially modulates both NK and NKT cells in systemic and mucosal organs relative to DSS. These results will need to be confirmed by a more focused study for any prudent conclusion to be drawn about the role of NK and NKT cells in the suppression of colitis.

### 3.10. DJ-X-013 induced infiltration of the colon with MDSCs and DCs

**Fig. 7.**
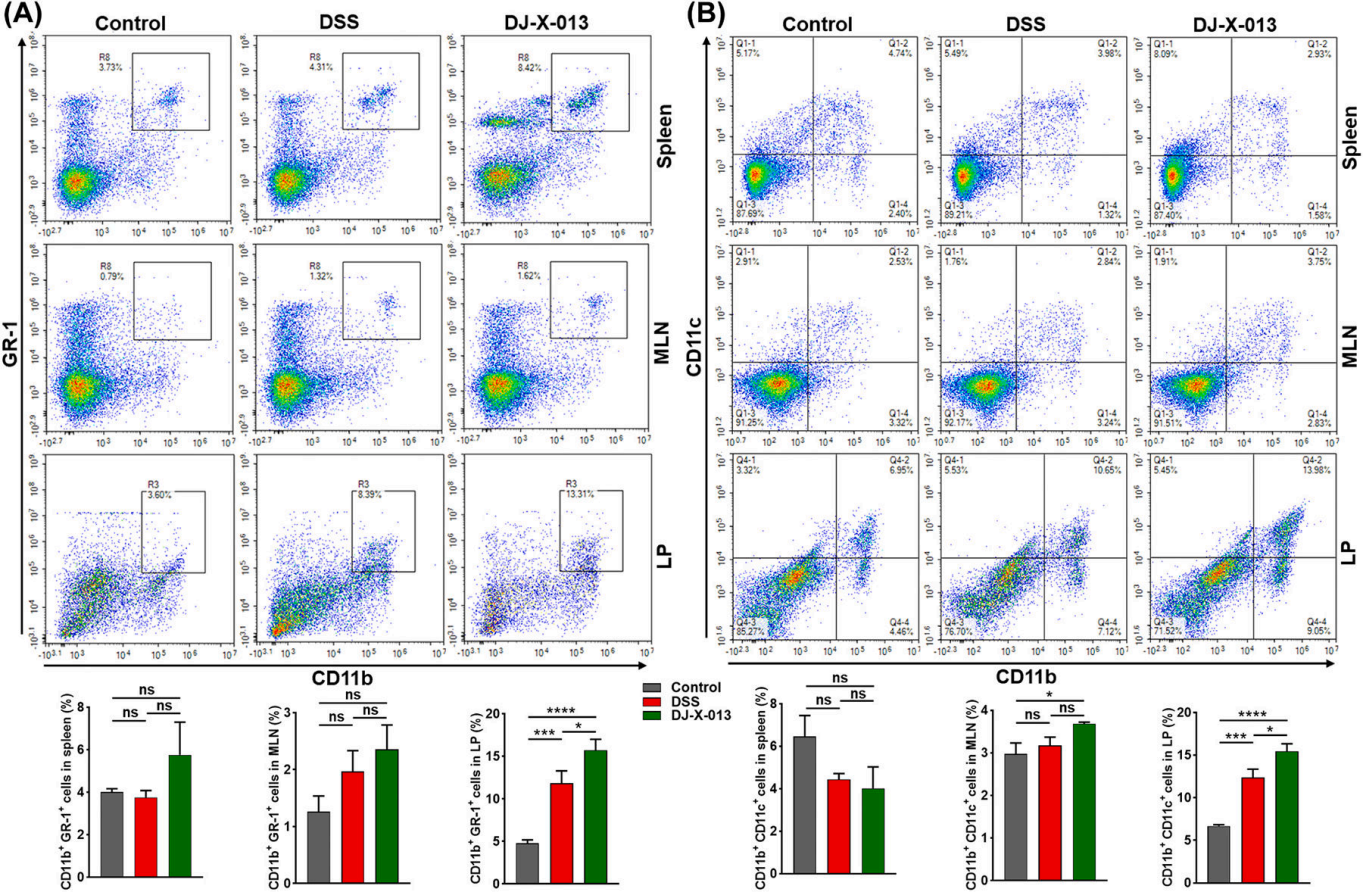
The effect of DJ-X-013 treatment in reducing colitis was mediated via MDSCs and dendritic cells. Spleens, MLNs, and colon LP immune cells were isolated from the three groups of mice (control, DSS, DSS + DJ-X-013) on day 8 and stained with antibodies specific for CD11b, GR-1, and CD11c. (A) The percentages of MDSCs (CD11b^+^ GR-1^+^) are shown in the upper right quadrants. (B) The percentages of dendritic cells (CD11b^+^ and CD11c^+^) are shown in the upper right quadrants. The representative density plot and related bar graph with analysis from repeated experiments are shown. Statistical analysis was performed using one-way ANOVA followed by Tukey’s multiple comparison post hoc tests; n = 6–9. Data are presented as mean values ± SEM, ns p > 0.05; *p < 0.05; ***p < 0.001; ****p < 0.0001.

MDSCs are a heterogeneous population of immature myeloid cells that serve as crucial players in the prevention and treatment of human diseases involving chronic inflammation [36]. T cells become activated during colitis as the result of efficient uptake and presentation of antigens to T-cells by dendritic cells (DCs) [49]. Thus, we assessed the effect of DJ-X-013 treatment on the frequency of systemic and mucosal MDSCs (CD11b^+^ GR-1^+^) and DCs (CD11b^+^ CD11c^+^) by flow cytometry. We observed that on induction of colitis in mice with DSS, the frequency of MDSCs was slightly increased in the MLNs, and significantly in colon LP, relative to those of control mice (boxed cells in Fig. 7A). Interestingly, treating mice with DSS and DJ-X-013 led to a prominent increase in frequency of MDSCs in colon LP and little in spleens, MLNs, relative to those after treatment with DSS alone (Fig. 7A in boxed cells). These results suggest that DJ-X-013 treatment induced the frequency of MDSCs, especially in LP, thereby protecting the colon from colitis.

We also evaluated the frequency of DCs frequency in control mice and those treated with DSS or DSS + DJ-X-013. We observed differential changes in the frequency of DCs in spleen, MLNs, and LP in mice treated with DSS and DJ-X-013 relative to those treated with DSS alone. The frequency of the splenic DCs slightly decreased in mice treated with DJ-X-013 vs DSS alone (Fig. 7B, upper right quadrants). In contrast, the numbers of DCs significantly increased colon LP and were not altered in the MLNs on treatment with DSS and DJ-X-013 relative to those detected on treatment with DSS alone (Fig. 7B, right quadrants). Taken together, these results propose that the DJ-X-013 have differential regulation of DCs in systemic and mucosal sites that helps to suppress colitis.

### 3.11. DJ-X-013 suppressed the frequency of inflammatory Th17 cells during colitis

**Fig. 8.**
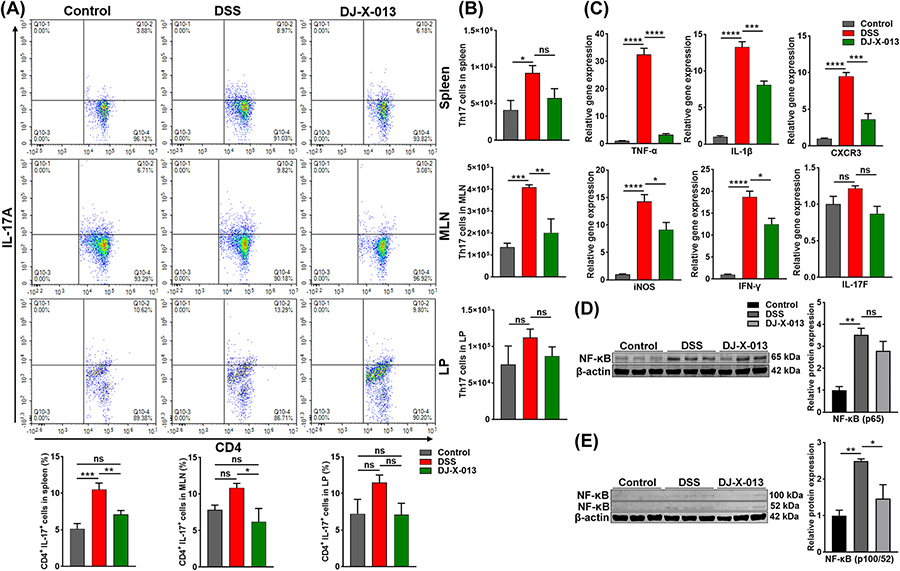
DJ-X-013 mitigated colitis symptoms by attenuating Th17 cells and inflammatory markers. (A) Spleens, MLNs, and colon LP immune cells were isolated from the three groups of mice (control, DSS, DSS + DJ-X-013) on day 8 and stained with antibodies specific for CD4 and IL-17A. The percentages of Th17 cells gated on CD4^+^ cells (CD4^+^ IL-17A^+^) are shown in the upper right quadrants. The representative density plot and related bar graph with analysis from repeated experiments are shown. Statistical analysis was performed using one-way ANOVA followed by Tukey’s multiple comparison post hoc tests; n = 6. (B) The actual total numbers of Th17 cells in the spleen, MLNs, and colon LP are shown. (C) The expression of mRNAs encoding pro-inflammatory TNF-α, iNOS, CXCR3, IL-1β, IL-17 F, and IFN-γ was examined in total RNA in colon tissue using RT-qPCR. (D) Images of representative immunoblots and their relative quantification of NF-kB (p65) in colon tissue. (E) Images of representative immunoblots and their relative quantification of non-canonical NF-kB (p100/52) in the colon tissue. One-way ANOVA followed by Dunnett’s post hoc test was applied in (B) n = 5 and (C-E) n = 3. Data are presented as mean ± SEM, ns p > 0.05, *p < 0.05, **p < 0.01, ***p < 0.001, ****p < 0.0001.

In both CD patients and experimental animal models of colitis, both the frequency of Th17 cells and the expression of IL-17 increase [25,50]. Since the role of IL-17 in intestinal inflammation remains controversial, we examined whether Th17 cells play any role in DSS-induced colitis and whether they would be affected by DJ-X-013 treatment. We used flow cytometry to show that the frequency of both systemic and mucosal CD4^+^ Th17 cells decreased after treatment of mice with DSS and DJ-X-013 relative to mice treated with DSS alone (Fig. 8A, upper right quadrant). We also determined that the number of Th17 cells increased in mice tested with DSS alone but decreased significantly particularly in spleen and MLN after treatment with DSS and DJ-X-013 (Fig. 8A and 8B). Thus, DJ-X-013 treatment appeared to modify the frequency and number of Th17 cells in mice with DSS-induced colitis, suggesting an inhibitory role on the Th17 cell response in DSS-induced colitis.

### 3.12. DJ-X-013 restricted the inflammatory response by targeting the NF-κB pathway in the DSS-induced model of colitis in mice

IBD patients exhibit high levels of activated NF-κB [51], which regulates many inflammatory markers [52]. Therefore, we measured the gene and protein expression of inflammatory mediators in colon tissue after treatment of mice with DSS and DJ-X-013 using RT-qPCR and immunoblot analysis. We observed that expression of mRNA encoding pro-inflammatory markers TNF-α, IL-1β, CXCR3, iNOS, and IFN-γ, as well as IL-17 F in the colon, was significantly reduced after mice were treated with DSS and DJ-X-013, relative to DSS alone (Fig. 8C). When we measured the accumulation of canonical and non-canonical NF-κB protein in colon tissue by immunoblot, we observed a reduction in NF-κB protein expression in the treatment of the mice with DSS and DJ-X-013, relative to DSS alone (Fig. 8D and 8E). We also observed that treatment of mice with DSS and DJ-X-013 suppressed the expression of systemic inflammatory cytokines and chemokines relative to the levels observed on treatment with DSS alone (Supplementary Fig. 3). Thus, taken together, compound DJ-X-013 appears to exert its anti-inflammatory action, at least in part, by repressing expression of the transcription factor NF-κB.

## 4. Discussion

While inflammation represents an immune system defense mechanism, prolonged inflammation can initiate diseases like inflammatory bowel disease (IBD). The available conventional therapies for IBD have a high incidence of relapse and are associated with adverse side effects like osteoporosis, myopathy, hypertension, weight loss, stomach upset, and increased risk of infection. Thus, there is an urgent need for safe, novel anti-inflammatory therapeutic options for IBD. We conceptualized and designed a nonsteroidal thiazole-based compound DJ-X-013 that predicted will share anti-inflammatory activity with other thiazole-based compounds, synthesized the compound, and have now assessed its effectiveness in ameliorating inflammation in a well-established DSS-induced model of colitis in mice. As a drug candidate, DJ-X-013 displayed excellent physical and drug-likeness properties, in particular high GI absorption, no penetration of the BBB, and high bioavailability. Here, we demonstrated that administration of DJ-X-013 reversed the severity of DSS-induced colitis in mice and reduced the LPS-induced inflammatory response in RAW264.7 macrophages. In the murine DSS-induced colitis model, administration of DJ-X-013 with DSS reversed weight loss, slightly lengthened the colon, and decreased the disease severity. DJ-X-013 treatment decreased the frequency of macrophage, activated T cells, neutrophils, and Th17 cells, induced MDSCs, differentially modulated NK, NKT, and DCs, and reduced expression levels of NF-κB associated with inflammation. Taken together, these results suggest that DJ-X-013 abrogates colitis by inducing MDSCs, suppressing the Th17 cells, and modulating NF-κB and cytoskeletal proteins, thereby reducing inflammatory response and colitis.

Macrophages are one of the important players during infection, inflammation, and autoimmune diseases [53,54], and serve as a sentinel for mucosal immunity in IBD [55]. We observed that LPS-induced RAW264.7 macrophages treated with LPS and DJ-X-013 maintained high cell viability at lower concentrations of DJ-X-013 and exhibited 50 % viability even at a 60 μM DJ-X-013 concentration. We showed that LPS stimulated the migration of RAW264.7 macrophages by altering their morphology from a round to an elongated shape, while treatment of LPS and DJ-X-013 restricted cell migration and reverted the cell morphology to a round shape. Since, cell shape and migration are generally regulated by polymerization, depolymerization, and interaction of cytoskeletal proteins like actin, tubulin, and microfilaments [56], we examined actin and tubulin organization in RAW264.7 macrophages using immunofluorescence confocal microscopy. The results of this experimental approach suggested that DJ-X-013 might have a modulatory role in the polymerization and depolymerization of these cytoskeletal proteins, an interpretation also consistent with changes in the cell morphology and migration potency observed in scratch assays after DJ-X-013 treatment. However, a more focused study on the role of DJ-X-013 in cytoskeletal protein polymerization/depolymerization will be required to draw a meaningful conclusion in this area. Furthermore, DJ-X-013 attenuated the inflammatory response of LPS-stimulated RAW264.7 macrophages, especially in the production of TNF-α and iNOS.

To determine the anti-inflammatory properties of DJ-X-013, we extended our *in vitro* work to the DSS-induced mouse model of colitis. The progression of experimental colitis is generally measured by a reduction in body weight and colon length [57]. We observed that treatment with DSS and DJ-X-013 improved body weight, showed a trend toward a slight increase in colon length, decreased splenomegaly, and reduced MLN’s inflammation in colitis, relative to the DSS group. Since, epithelial damage, submucosal hypertrophy with thickening due to edema, and disorganized collagen have been reported in IBD and colitis [58,59]. Interestingly, the DJ-X-013 compound appears to have facilitated colon epithelial healing, restored submucosal architecture and reduced the infiltration of inflammatory cells in the colon of mice with DSS-induced colitis. This improvement of colitis symptoms was mirrored in the reduced inflammation score observed in the DSS and DJ-X-013 treated group relative to the group that received DSS alone. Detailed histological changes in the colon after DJ-X-013 treatment validated the reduction of inflammation score and histomorphological alterations. The decrease in inflammation was further endorsed by extensive analysis of immune cells in the colon LP, spleen, and MLNs by flow cytometry.

Activated T cells, macrophages, and neutrophils are positively associated with the inflammation that accompanies IBD pathogenesis [60,61], and colitis is characterized by a higher influx of activated CD4 T cells and Th17 cells [10,62]. When we examined the alterations in macrophage, neutrophils, and Th17 cells after treatment of mice with DSS and DJ-X-013, we observed restricted infiltration of systemic and colon neutrophils and Th17 cells relative to those observed in mice treated with DSS alone. We also observed specific inhibition of TNF-α producing macrophages and activated monocytes and impaired homing activation of CD4^+^CXCR3^+^ T cells in the colon of mice treated with DSS and DJ-X-013 compared to DSS alone. Since we and others have shown that reduction in the numbers of activated T cells, macrophages, Th17 cells, and neutrophils are the hallmarks of colitis abrogation [63–65], our data suggest that DJ-X-013 suppressed colitis in part by reducing the number of activated macrophages, T cells, neutrophils, and Th17 cells in the colon.

Mounting evidence shows that MDSCs (CD11b^+^ GR1^+^) improve colon healing in DSS-induced colitis by inhibiting the infiltration of inflammatory cells, especially macrophages and activated T cells [37,66], and inducing MDSCs leads to suppression of colitis [32,67,68]. We observed that DJ-X-013 exhibited strong anti-inflammatory properties against both mucosal and systemic inflammation in DSS-induced colitis. Interestingly, we also observed that the percentage of MDSCs increased in the colon of mice treated with DSS and DJ-X-013 compared to DSS alone. Thus, our study has determined that in the DSS-induced mouse model of colitis, DJ-X-013 treatment improves inflammation by inducing MDSCs, which suppress the inflammatory response of immune cells including neutrophils, TNF-α producing macrophages, monocytes, and activated T cells, and inhibit migration of Th17 cells in the colon LP and systemic organs. Thus, the regulation of several pro-inflammatory cells and cytokines by MDSCs might elicit suppressive functions in colitis.

The potential roles of DCs in IBD are controversial and depend on DC subtypes, including conventional (cDC) and plasmacytoid (pDC) DCs [69,70]. Furthermore, DCs form a network that regulates intestinal inflammation and immune tolerance to symbiotic bacteria [71]. While natural killer (NK) cells have differential effects in autoimmune diseases, there is mounting evidence that these cells induce IBD pathogenesis [72]. Therefore, we examined the ability of DJ-X-013 to mediate DCs, NK, and NKT cells in DSS-induced colitis. We observed that DCs (CD11b^+^ CD11c^+^) were elevated in the colon of the group treated with DSS and DJ-X-013 relative to the group treated with DSS alone. Thus, our data suggest that DJ-X-013 may induce the function of DCs and might play a role in reducing colon inflammation. We also observed a reduction in the NK cell population in the colon of the mice treated with DSS and DJ-X-013 and an increase in their numbers in systemic organs like spleen and MLN relative to those treated with DSS alone. Further, NKT cells are known to either boost or inhibit cellular immunity in various pathological disorders [73], serve regulatory roles in autoimmune diseases, and suppress Th17 cells [74]. Here we observed that NKT cells were increased in the mucosal and systemic immune organs of the DSS + DJ-X-013 group as compared to the group that received DSS alone. Thus, taken together, our study suggests that DJ-X-013 exhibits protective roles in colitis by differentially modulating DCs, NK, and NKT cells, which may in part inhibit Th17 cells and reduce the inflammatory response in DSS-induced colitis.

The transcription factor NF-κB serves as a master regulator of inflammation-induced cytokines that contribute to the stimulation and activation of immune cells, while inhibition of NF-κB activity has been strongly suggested as an anti-inflammatory strategy for the treatment of many diseases that are characterized by inflammation [75]. NF-κB is highly activated in IBD patients [51] and is associated with the macrophage-specific inflammatory response [76]. We extended our study to the molecular level by validating the expression of mRNAs encoding inflammatory markers and NF-κB by RT-qPCR and the corresponding proteins by immunoblot in colon tissue from mice treated with DSS and DJ-X-013. We observed attenuated expression of inflammatory cytokines like TNF-α, IL-1β, IFN-γ, iNOS, and CXCR3 and reduced expression of NF-κB, which is intricately associated with the activation of those inflammatory markers.

## 5. Conclusion

In summary, we conclude that DJ-X-013 exhibits potent anti-inflammatory functional properties at the cellular and molecular levels that affect three inter-linked routes, namely; **i**) inducing the frequency of MDSCs in the colon; **ii**) attenuating the expression of inflammatory markers like TNF-α, IL-1β, and NF-κB; and **iii**) reducing numbers of Th17 cells, macrophage, neutrophils, and activated T cells. DJ-X-013 may also in part restrict the migration of inflammatory cells to the colon LP by modulating cytoskeleton proteins, although the latter observation will need to be confirmed by further in-depth study.

## Supplementary Material

mmc2

mmc1

mmc3

## Figures and Tables

**Table 1:** List of anti-mouse antibodies used for flow cytometry.

Name of the protein	BioLegend Cat. No.^a^	Conjugated fluorophore	Concentration used for 10^6^ cells	Host

TNF-α	506308	APC	0.25 μg	Rat
iNOS	696806	PE	0.125 μg	Rat
CD11b	101208	PE	0.25 μg	Rat
CD11c	117311	AF488	0.25 μg	Armenian Hamster
Gr-1	108412	APC	0.25 μg	Rat
Ly6C	128046	APC Fire 750	0.5 μg	Rat
CD3	100204	FITC	1 μg	Rat
CD4	100412	APC	0.25 μg	Rat
CXCR3	126506	PE	0.25 μg	Armenian Hamster
NK1.1	108708	PE	0.25 μg	Mouse
IL17A	506926	BV421	0.25 μg	Rat

Abbreviations used: AF, Alexa fluor; APC, Allophycocyanin; BV, Brilliant violet; CD, Cluster of differentiation; CXCR3, CXC motif chemokine receptor 3; FITC, Fluorescein isothiocyanate; Gr-1, glutathione reductase; IL, Interleukin; iNOS, Inducible nitric oxide synthase; Ly6C, lymphocyte antigen 6 family member C1; NK1.1, Killer cell lectin-like receptor subfamily B member 1 C; PE, Phycoery-thrin; TNF-α, Tumor necrosis factor-alpha.

a Antibodies used for flow cytometry were purchased from BioLegend, Inc. (San Diego, CA, USA).

**Table 2:** List of primer sequences used for RT-qPCR.

Gene Name	Sequence (5’ to 3’)	

*Gapdh*	For	GAAGCCCATCACCATCTT
	Rev	CAGTAGACTCCACGACATAC
*Tnfa*	For	GGTGCCTATGTCTCAGCCTCT T
	Rev	GCCATAGAACTGATGAGAGGGAG
*iNos*	For	GGAGAGAGATCCGATTTAGAG
	Rev	CCTGTCTCAGTAGCAAAGA
*Il6*	For	CAGAGTCCTTCAGAGAGATAC
	Rev	ATGGTCTTGGTCCTTAGC
*Il1b*	For	TGGACCTTCCAGGATGAGGACA
	Rev	GTTCATCTCGGAGCCTGTAGTG
*Nfkb*	For	GAAGACACGAGGCTACAA
	Rev	GGAAGGCATTGTTCAGTATC
*Stat3*	For	AGGAGTCTAACAACGGCAGCCT
	Rev	GTGGTACACCTCAGTCTCGAAG
*Cxcr3*	For	GCTTGTCCTCCTTGTAGT
	Rev	GTCCTTGTTGCTGAGATTG
*Ifng*	For	GACCTAGAGAAGACACATCAG
	Rev	AACAGCCATGAGGAAGAG
*Il17f*	For	CCCAATGCCTCACTTAAAC
	Rev	CAAGAACTGTGGTCATCATC

a Primers were purchased from IDT, Coralville, IA, USA.

Abbreviations used: CXCR3, CXC motif chemokine receptor 3; For, forward; Gapdh, Glyceraldehyde-3-phosphate dehydrogenase; IFNg; Interferon gamma; IL, Interleukin; iNOS, Inducible nitric oxide synthase; Nfkb, Nuclear factor kappa-light-chain-enhancer of B cells; Rev, reverse; STAT, Signal transducer and activator of transcription; TNFa, Tumor necrosis factor-alpha.

**Table 3:** Predicted physicochemical, pharmacokinetic, and drug-likeness properties of DJ-X-013.^a^.

DJ-X-013	**Physicochemical parameters**					
	Formula	MW (g/mol)	HA	RB	HBA	HBD
	C_19_H_19_NO_4_S	357.42	25	6	5	1
Structure	**Pharmacokinetic parameters**				
	MLOGP		GI		BBB	
			absorption		permeation	
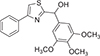	1.29		High		No	
	**Drug-likeness properties**					
	Lipinski’s Rules of Five			Bioavailability		
	Yes: 0 violations			0.55			

a Properties were determined using SwissADME predictive software [39].

Abbreviations used: BBB, Blood-brain barrier; GI, Gastrointestinal; HA, Number of heavy atoms; HBA, Number of hydrogen-bond acceptors; HBD: Number of H-bond donors; MLOGP, Moriguchi octanol-water partition coefficient (LogP_o/w_ topological method); MW, Molecular weight; RB, Number of rotatable bonds.

## Data Availability

Data will be made available on request.

## Abbreviations:

ADME: Absorption distribution, metabolism and excretion
AKT: Rac-alpha serine/threonine-protein kinase
ANOVA: Analysis of variance
BBB: Blood-brain barrier
CD: Crohn’s disease
CREB: cAMP response element-binding protein
CXCR3: C-X-C Motif Chemokine Receptor 3
DAPI: 4,6-diamidino-2-phenylindole
DC: dendritic cell
DMEM: Dulbecco’s modified essential medium
DMSO: dimethyl sulfoxide
DSS: dextran sodium sulfate
ERK/MAK: Extracellular signal-regulated kinase/Mitogen-activated kinase
FBS: Fetal bovine serum
GI: Gastrointestinal
H&E: hematoxylin and eosin stain
HA: Number of heavy atoms
HBA: Number of hydrogen-bond acceptors
HBSS: Hanks’ balanced salt solution
HIA: Human intestinal absorption
IB: Immunoblot
IBD: Inflammatory bowel disease
IF: Immunofluorescence
IFN-γ: Interferon gamma
IL: Interleukin
INOS: Inducible nitric oxide synthase
JNK: cJun N-terminal kinase
LP: Lamina propria
LPS: Lipopolysaccharide
MDSC: Myeloid-derived suppressor cell
MFI: Mean fluorescence intensity
MLN: Mesenteric lymph node
MLOGP: Moriguchi octanol-water partition coefficient
MT: Microtubule
MTT: 3-[4,5-dimethylthiazol-2-yl]-2,5 diphenyl tetrazolium bromide
MW: Molecular weight
NF-κB: Nuclear factor kappa-light-chain-enhancer of activated B cells
NK cell: Natural killer cell
NKT: Natural killer T cells
PBS: Phosphate-buffered saline
RB: Number of rotatable bonds
RT-qPCR: Reverse transcription-quantitative polymerase chain reaction
RPMI medium: Roswell Park Memorial Institute Medium
RT: Room temperature
SEM: Standard error of the mean
STAT: Signal transducer and activator of transcription
Th cell: T helper cell
TNF-α: Tumor necrosis factor alpha
UC: Ulcerative colitis
