# Supplementary figures and images for: DJ-X-013 reduces LPS-induced inflammation, modulates Th17/myeloid-derived suppressor cells, and alters NF-κB expression to ameliorate experimental colitis
Source: Biomed Pharmacother. Author manuscript; Available in PMC 2024 Oct 15. (PMC11479677; doi:10.1016/j.biopha.2024.117379)

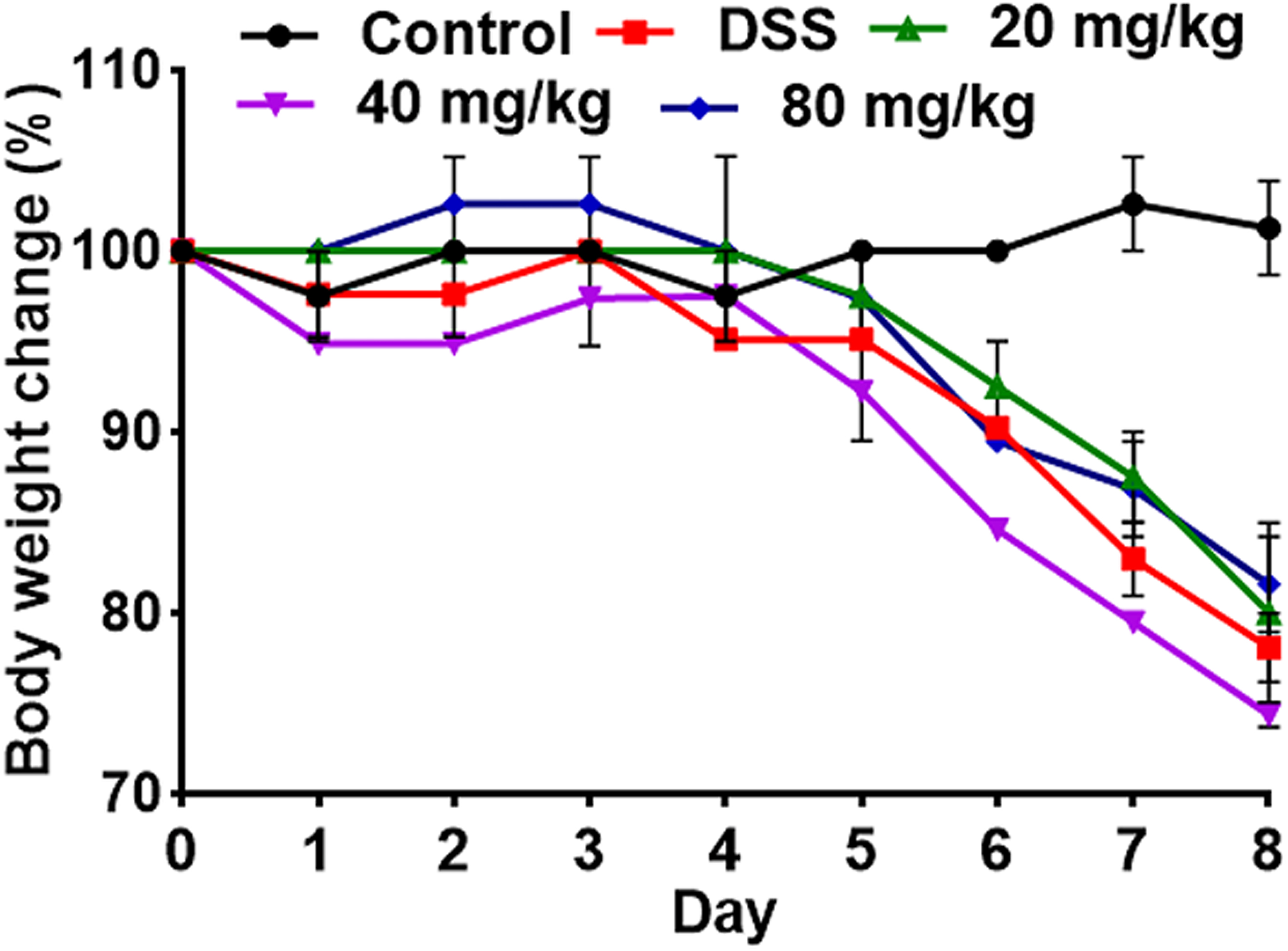

Supplement: mmc2 [file NIHMS2024395-supplement-mmc2.jpg]

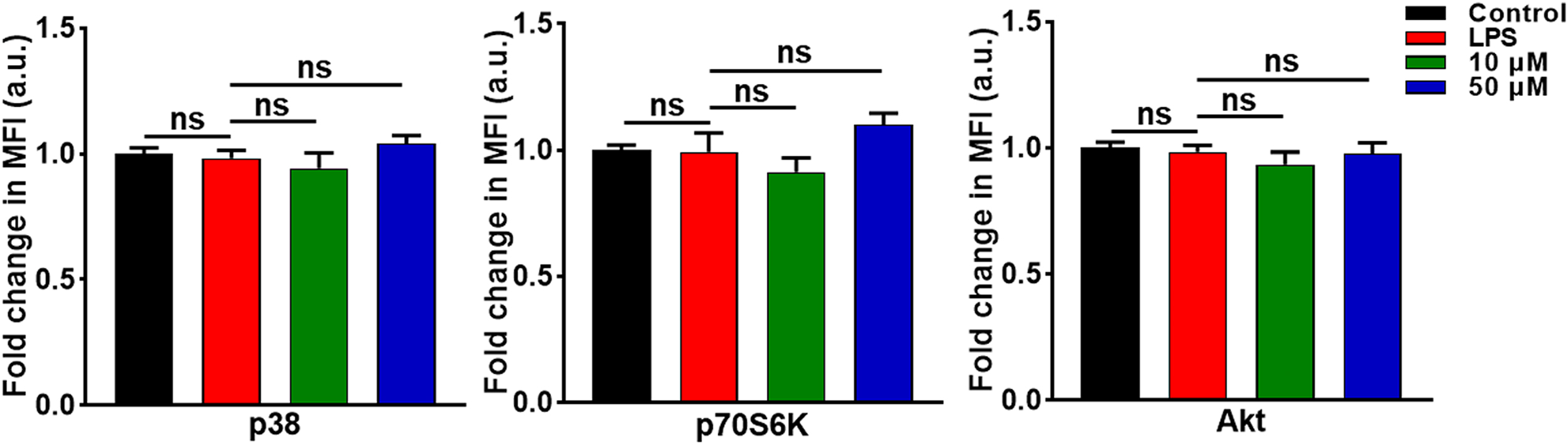

Supplement: mmc1 [file NIHMS2024395-supplement-mmc1.jpg]

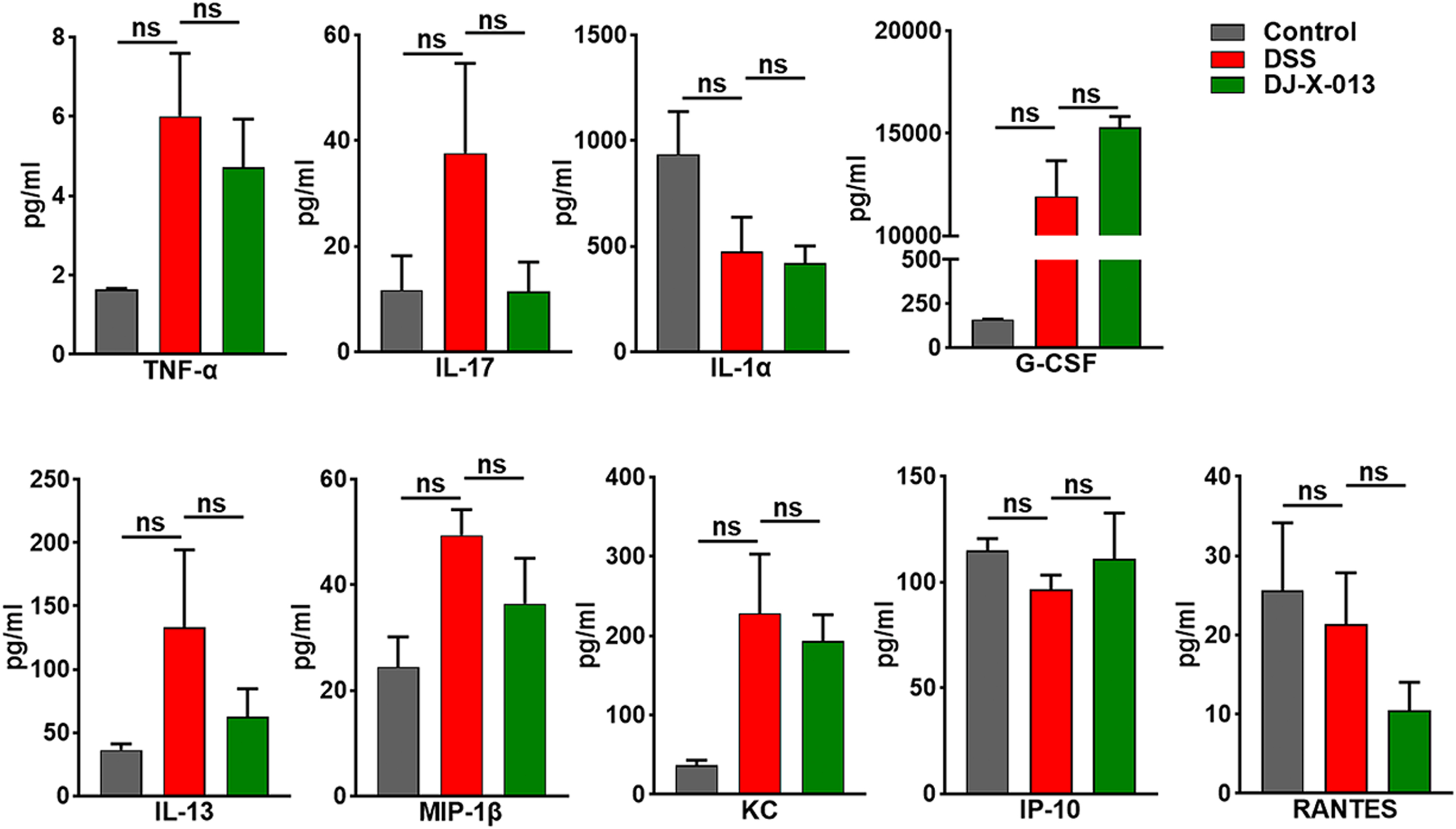

Supplement: mmc3 [file NIHMS2024395-supplement-mmc3.jpg]
